# 3DOpt: Benchmark for Automated Design of 3D Molecular Structures across the Periodic Table

**DOI:** 10.1021/acs.jcim.6c00259

**Published:** 2026-05-19

**Authors:** Marcello Costamagna, Morgan Thomas, Marco Foscato, Vidar R. Jensen

**Affiliations:** † Department of Chemistry, 1658 University of Bergen, Allégaten 41, Bergen N-5007, Norway; ‡ Computational Science Laboratory, Universitat Pompeu Fabra, PRBB, Barcelona 08003, Spain

## Abstract

The automated design of three-dimensional (3D) molecular structures is a rapidly advancing field with major applications in drug discovery, catalysis, and materials science. Despite this progress, there remains a lack of standardized benchmarks for objectively evaluating and comparing generative methods for 3D molecular design beyond the domain of organic drug-like compounds. Here, we present 3DOpt, the first benchmark designed to assess the ability of generative methods to identify optimal 3D structures across the full chemical spectrum, including organometallic and transition-metal complexes. Each benchmark task is defined by a target 3D structure, a rigorously curated starting population of pre-evaluated candidate molecules, and a scoring function that quantifies similarity to the target in terms of both geometry and composition. We demonstrate the utility of 3DOpt by applying baseline generative methods and provide reference performance metrics for widely used molecular design strategies. Overall, 3DOpt establishes a general-purpose framework for the systematic evaluation of 3D molecular generative design methods across diverse chemical spaces.

## Graphical abstract

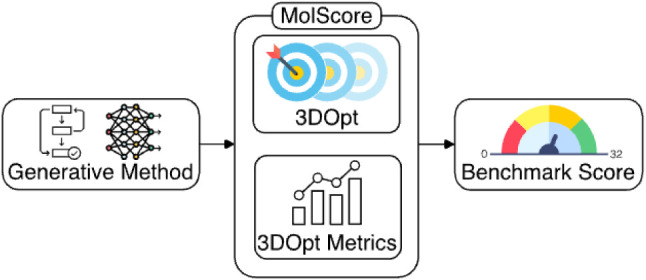

## Introduction

Molecular design aims to identify molecules with specific desired properties from a vast pool of possible candidates. Unsurprisingly, the field has been significantly advanced by computational methods capable of predicting the properties of molecules proposed by human experts.^1,2^ Today, the design of candidate molecules itself can be automated using generative methods—computer algorithms that, given a definition of desired molecular properties, produce candidate molecules expected to exhibit those properties.^3,4^ The development of such methods is considered a solution to the inverse molecular design problem^5,6^ and is referred to as automated de novo in silico design, often shortened to de novo design.^7^ Originating in the early 1990s,^8–11^ de novo design has evolved substantially, particularly within the drug design community, leading to a range of methods that have achieved remarkable success,^12,13^ as confirmed by the synthesis and validation of de novo-designed pharmaceuticals.^14,15^ While experimental validation remains the ultimate proof of real-world utility, synthesis and laboratory testing are inherently time-consuming, expensive, and resource-intensive. Consequently, experimental validation is not a feasible routine practice for prospective comparison, especially given the large number of existing de novo design methods and the rapid pace of new developments, recently accelerated by advances in artificial intelligence.^16^

The challenge of comparing generative methods is therefore addressed through the use of virtual benchmarks—systematic and reproducible evaluation frameworks for comparing the performance of generative methods without experimental validation.^17–19^ Beyond serving as standardized proxies for experimental testing, benchmarks have proven to be powerful drivers of innovation in other domains, including image recognition,^20,21^ natural language processing,^22^ and materials design.^23^ In these fields, benchmarks have helped align research goals, refine evaluation metrics, accelerate methodological progress, and inform the application of generative approaches.^21,24–26^

Driven by the needs of organic drug design, the first benchmark for de novo molecular design, GuacaMol,^18^ introduced two suites of tests: distribution-learning benchmarks, which evaluate the ability to generate a given distribution of molecules, and goal-directed benchmarks, which assess a method’s ability to optimize molecules toward specific objectives. Shortly thereafter, MOSES^27^ focused exclusively on distribution-learning methods and introduced new performance metrics, sparking a broader discussion that led to further developments (Figure 1).

**Figure 1:**
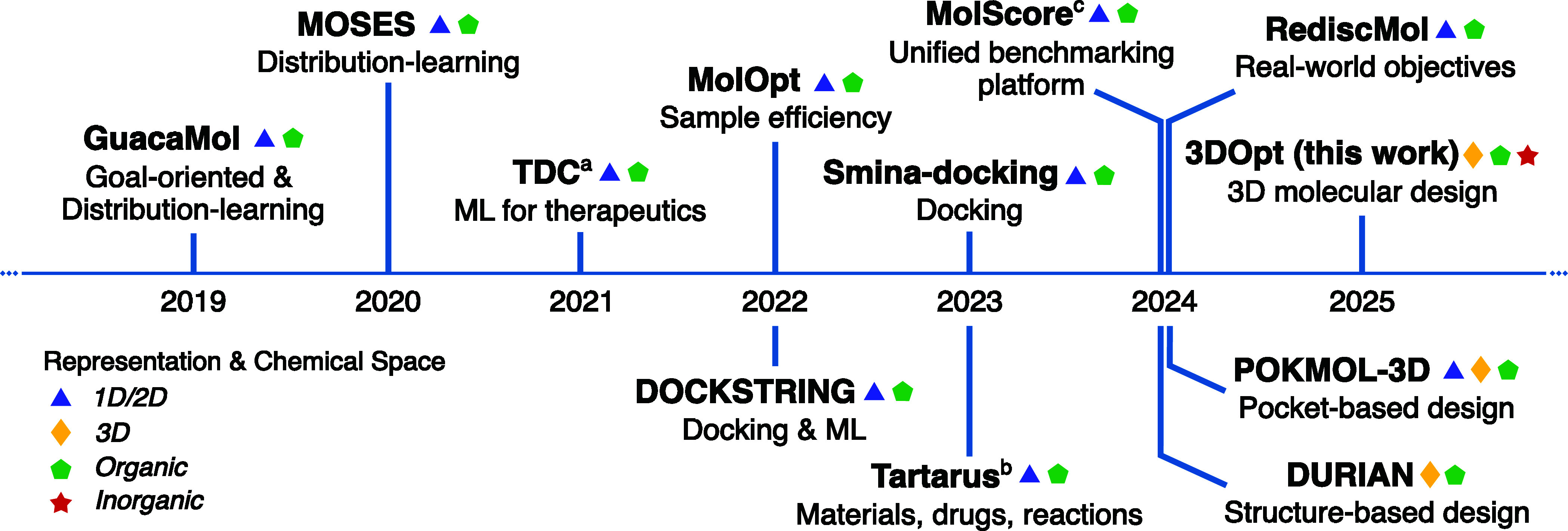
Timeline of de novo molecular design benchmarks from GuacaMol to 3DOpt (this work). Each benchmark is annotated with its primary focus. ^a^Platform to evaluate machine learning methods across drug discovery tasks. ^b^Framework to assess molecular design for drugs, materials, and chemical reactions. ^c^Unified platform for running and customizing multiple benchmarks and scoring functions. ^d^refs. 32–40 .

Early critiques of these initial benchmarks highlighted three major shortcomings:^28,29^ oversimplified single-property objectives, the lack of tests for real-world applicability, and the absence of budget-aware sample-efficiency measures (i.e., tracking how many molecules are tested to produce a sample of a given quality). Subsequent benchmarks have sought to address these limitations. For example, MolOpt^30^ enforces rigorous, budget-constrained evaluations of sample efficiency, while RediscMol^31^ advances realism by replacing heuristic property scores with a biologically grounded objective—namely, the ability to rediscover experimentally confirmed actives.

To address the specific challenges inherent to molecular docking, tailored benchmarking approaches, such as DOCKSTRING^41^ and Smina-docking,^42^ have been developed. In parallel, the Therapeutics Data Commons (TDC)^43^ initiative has provided a versatile platform for evaluating the growing number of machine-learning (ML) methods applied across drug discovery. Among its tools, TDC includes a customizable reimplementation of GuacaMol along with a suite of evaluation metrics for drug design. Expanding beyond drug design, Tartarus^44^ was introduced to benchmark ML models in the molecular design of organic materials, pharmaceuticals, and chemical reactions. In 2024, many of these benchmarks, along with their associated metrics, scoring functions, and additional functionalities, were consolidated into a unified package named MolScore.^45^ MolScore streamlines access to these resources and significantly lowers the barrier to creating new benchmarks, allowing users to build custom workflows by leveraging existing tools or integrating novel components.

Collectively (Figure 1), the benchmarks discussed above reflect the historical emphasis of de novo design methods on small organic molecules represented in one or two dimensions, that is, string- and graph-based representations, most commonly via SMILES.^46,47^ As noted by Thomas et al.,^45^ however, the growing interest in methods for generating three-dimensional (3D) molecular structures^48,49^ necessitates an expansion of benchmarking frameworks to accommodate 3D molecular representations. These 3D generative approaches aim to produce full molecular geometries optimized with respect to objective functions that depend on spatial structure, rather than solely on atomic identity and connectivity.

In contrast, de novo design has historically relied heavily on low-dimensional molecular representations, such as SMILES,^46,47^ SELFIES,^50^ and molecular graphs,^51^ hereafter termed 1D/2D representations. These formats are computationally efficient and have proven effective for many tasks involving small organic compounds governed primarily by valence rules.^52,53^ Because of their efficiency, several strategies have been proposed to extend their applicability to contexts where 3D-dependent properties are critical. Some approaches introduce modified 1D/2D representations enriched with additional features to enhance predictive accuracy,^54–57^ while others attempt to infer 3D-dependent properties directly from these simplified formats.^58,59^

While all molecular properties are, in principle, encoded in the constitution of a system, as defined by atomic composition and connectivity, many practically relevant properties, such as binding affinities^60,61^ or quantum descriptors,^62–64^ depend subtly on the 3D geometry and cannot yet be efficiently and reliably inferred without explicit geometrical information. Thus, regardless of the initial representation, most workflows for evaluating 3D-dependent properties ultimately require tools for generating plausible 3D structures,^65^ that is, one or more conformers, to enable meaningful property prediction. This dependency renders the accuracy and reliability of conformer generation a critical bottleneck in the de novo design of 3D structures.

Conformer generation is a well-established field with a wide array of available tools and methods.^66–70^ Most existing approaches are accurate and robust for small, drug-like organic molecules, reflecting their origins in pharmaceutical and medicinal chemistry.^65,70–76^ Only a few methods explicitly accommodate inorganic or organometallic compounds.^77^ Traditional algorithms often rely on distance-matrix heuristics or hand-crafted force fields, which can limit both the accuracy of resulting geometries and the efficiency of conformational sampling.^65,78–81^ Recent ML approaches aim to overcome these limitations by learning molecular energy landscapes or torsional preferences directly from data, thereby improving both speed and predictive fidelity.^78–82^ Some efforts have extended coverage to transition metal complexes. For example, *Architector* supports mononuclear complexes across the periodic table.^83^ Nonetheless, significant challenges persist in the conformer generation of metal-containing systems, particularly in handling polynuclear complexes and multihapto ligation.^83^ Compounding these difficulties is the scarcity of reliable, comprehensive data sets for metal-containing molecules.^83,84^ Furthermore, representational limitations pose a major obstacle: conventional 1D/2D formats fail to capture key features such as coordination environments, oxidation states, and nonclassical bonding patterns.^85^ As a result, these representations systematically struggle to encode metal-centered stereochemistry, atom hybridization, aromaticity, implicit hydrogen counts, and bonding motifs such as dative and multihapto interactions, metal hydrides, and high coordination numbers—features that are essential for realistic modeling of inorganic and metal-containing molecules.

Given the critical roles of inorganic molecules in catalysis,^86–91^ functional materials,^92–96^ and metallodrugs,^97–100^ there is growing interest in overcoming the limitations of conventional 1D/2D representations. To address these challenges, researchers have increasingly turned to methods that operate directly in 3D space for both organic^48,101^ and inorganic^86,93,102,103^ molecules. The proliferation of 3D generative approaches has, in turn, spurred the development of benchmarking frameworks tailored to structure-based drug design (SBDD). In recent years, several such benchmarks have been introduced, including DURIAN,^32^ POKMOL-3D,^33^ DrugPose,^34^ GenBench3D,^35^ and CBGBench,^36^ alongside other related efforts.^37–40^ These benchmarks primarily evaluate generative models, particularly ML-based ones,^104–106^ with an emphasis on producing valid molecular structures that adopt conformations favorable for protein binding.

However, no benchmark currently exists for evaluating generative methods based solely on the 3D structures of the generated molecules independent of any protein target or chemical context. Moreover, existing benchmarks do not consider organometallic or metal-containing compounds, which present some of the most challenging structural motifs in chemistry. These include high coordination numbers and unconventional bonding patterns, such as dative and multihapto interactions, that are difficult to represent accurately using non-3D chemical formats.

To address these shortcomings, we present *3DOpt*, the first benchmark designed to evaluate the capabilities of generative methods for optimizing 3D molecular structures across both organic and inorganic compounds. 3DOpt is integrated into the forthcoming version (already available as v2.0.0-beta)^107^ of the MolScore platform, which has been specifically updated to support 3D chemical representations.

## Methods

The 3DOpt benchmark provides a comprehensive, extensible, and reproducible framework for evaluating generative methods on the challenging task of constructing realistic three-dimensional (3D) molecular structures across both organic and inorganic compounds. Developed as an integral part of the modular MolScore platform, 3DOpt comprises a suite of 32 rediscovery tasks. The underlying principle is straightforward: if a method can systematically “rediscover” a hidden target 3D structure starting from a structurally distant initial population, it demonstrates the ability either to generate plausible 3D structures or, given the opportunity to evaluate new candidates, to navigate the structural space toward the target. The difficulty of each task is determined by the structural complexity of the target and the degree of dissimilarity between the target and the starting population, which may or may not contain key structural motifs present in the target. The rediscovery tasks in 3DOpt span a wide range of atomic geometries and bonding patterns, challenging generative methods well beyond conventional organic-only chemical space.

In summary, each task defines (i) a specific target molecule to be rediscovered, which remains unknown to the generative method under evaluation; (ii) an initial set of scored structures (i.e., the starting population), representing the task-specific input provided to the generative algorithm; and (iii) a scoring function that enables the method to convert candidate 3D molecular representations into a figure of merit relevant to the design objective.

In the following sections, we first describe the selection and curation of the molecular structure data sets used to define each of the 32 rediscovery tasks. We then outline the principles guiding the selection of targets and starting populations as well as the baseline generative methods used to establish minimal performance within the 3DOpt benchmark. Finally, we present the benchmark metrics that summarize method performance across tasks and describe the implementation of 3DOpt within the MolScore platform.

### Data Set Generation

The Cambridge Structural Database (CSD)^108,109^ was selected as the source of 3D molecular structures used as targets or starting populations due to its comprehensive coverage of both organic and inorganic compounds. While this choice enhances the realism and relevance of the benchmark, it also introduces licensing constraints: access to 3D structures from the CSD, such as via the CSD Python API, requires a valid CSD license.

The CSD was filtered to select structures relevant for the benchmark tasks based on the following criteria: (i) presence of 3D coordinates; (ii) nonpolymeric nature; (iii) absence of disordered atoms; (iv) completeness of the 3D structure, including hydrogen atoms;^110^ and (v) availability of a canonical SMILES via the CSD Python API. Since many CSD entries contain multiple molecular entities (components), such as counterions and solvents, we implemented a procedure to select a single component per entry. The selection was based on three criteria: (i) largest molecular weight; (ii) highest atom count; and (iii) organometallic character, defined according to CSD conventions as containing both carbon and at least one metal atom from the transition metals, p-block metals, lanthanides, or actinides (excluding s-block elements). For each entry, we selected the first component that satisfied at least two of these three conditions and contained more than four atoms. Further details about the filtering pipeline are provided in (Section S3.2 of the Supporting Information SI).

Filtering the CSD release 2024.4 yielded a subset of 868 992 entries, hereafter referred to as the viable structures, which serves as the pool for selecting targets and generating the starting populations, as described in the following sections.

### Targets

The choice of target molecules determines the scope and complexity of the benchmark tasks. To balance chemical relevance and diversity, we adopted a principled selection strategy (details in Section S1 of the Supporting Information). Chemical relevance was ensured by sourcing exclusively from the Cambridge Structural Database (CSD), which contains experimentally determined structures.^110^ To promote diversity, we manually searched the CSD for molecules representing a broad range of connectivity patterns. Automated queries were not feasible for this purpose (see below). Importantly, we avoided selecting the most common connectivity motifs; while these would reflect the current CSD distribution, they would not sufficiently challenge generative methods with rare, yet experimentally validated patterns.

To guide target selection, we focused on the diversity of atom connectivity patterns arising from formal two- or three-center bond components irrespective of element identity. Ignoring element identity is essential because the number of possible element combinations grows rapidly. For instance, even the simplest two-atom connection yields N­(N+1)/2 combinations, where N is the number of elements. Considering the entire periodic table (*N* > 100), even restricting to experimentally observed combinations would require sampling an impractically large number of targets, making such an approach unsuitable for benchmarking.

Even when ignoring element identity, we are not aware of any existing topological characterization method that can classify formal atom connectivity patterns without two major limitations: reliance on fingerprints^111^ derived from predefined connectivity definitions (restricted to a fixed set of bond types)^112,113^ or the need for detailed electronic-state characterization, which inherently depends on geometry and element identity.^85,114–117^

To address this gap, we introduce the concept of a Basic Connectivity Pattern (BCP), defined as an element-independent pair or triad of atoms connected by a bond resulting from electron delocalization over those atoms. Each BCP is further characterized by (i) the symmetry (σ or π) of the bonding molecular orbital, and (ii) the cyclicity, i.e., whether at least two atoms of the BCP are embedded in a cycle. The combination of these three features, the number of atoms, orbital symmetry, and cyclicity, defines eight distinct BCPs that capture all possible σ- or π-type connectivity patterns irrespective of element identity (Figure S1, Supporting Information). This framework naturally accommodates nonstandard bonding motifs such as dative, multihapto, and three-center two-electron (*3c–2e*) bonds,^118^ which are rarely annotated explicitly in standard chemical representations.

Figure 2 illustrates one such case: the multihapto coordination of an olefin resulting from σ-donation of electrons from a π-bond between two atoms to a third atom. This interaction is complemented by π-back-donation from the third atom into the π* orbital of the olefin. Although these bonding motifs are only implicit in conventional chemical representations, both interactions are captured within the BCP formalism: BCP-5 for σ-donation, and BCP-7 for π-back-donation (see Figure 2).

**Figure 2:**
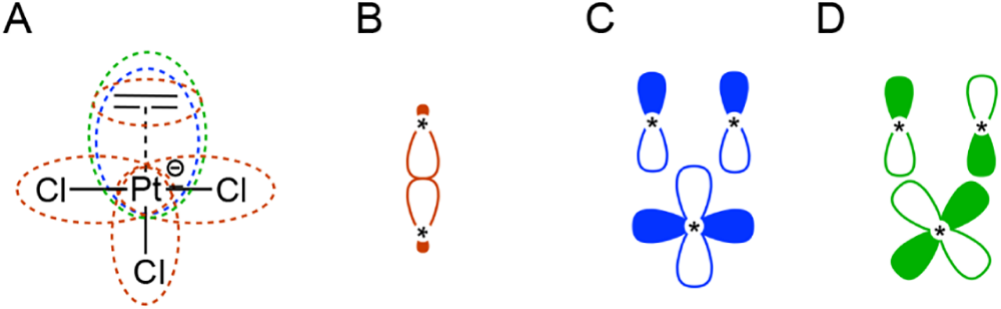
Three of the Basic Connectivity Patterns (BCPs) defined in Section S1. (A) Molecule containing the distinct BCPs: BCP-1 (red ellipses), BCP-5 (blue ellipse), and BCP-7 (green ellipse) exemplified in (B), (C), and (D), respectively, with atomic orbital components relevant for the compound in (A). (B) BCP-1: two-center two-electron (*2c–2e*) bond with σ symmetry with respect to the bond axis between the atoms (any atom is represented by an asterisk). (C) BCP-5: three-center two-electron (*3c–2e*) bond with σ symmetry. (D) BCP-7: three-center two-electron (*3c–2e*) bond with π symmetry. C–H bonds (BCP-1) are omitted for clarity.

Because these connectivity patterns cannot be readily expressed using SMARTS or related query languages, BCPs were defined textually and used as a visual guide to help chemists identify the smallest connectivity components within molecules (see Figure 2). The resulting set of BCPs informed our manual target selection and ensured that the final benchmark spanned a diverse range of connectivity patterns. Additional details on the definition and use of BCPs are provided in Section S1 of the Supporting Information.

In addition to ensuring diversity in connectivity patterns, we restricted the selection to molecules with limited conformational flexibility by selecting targets with few rotatable bonds. This criterion minimizes discrepancies between the solid-state geometries reported in the CSD and the dominant conformers in the solution or gas phase that generative methods might produce.

On the basis of these criteria, we selected 32 target molecules: 6 organic and 26 inorganic structures. Their CSD identifiers, 2D representations, and annotations highlighting relevant structural features are provided in Section S1.3 of the Supporting Information.

### Scoring Functions

The benchmark tasks in 3DOpt evaluate each generated molecular structure using 3D-aware molecular similarity to the task-specific target as a figure of merit. Similarity is computed using the Hypershape Recognition (HSR) package,^119^ which generates a molecular fingerprint encoding both the 3D geometry and selected atomic features. This approach enables fast, general, and scalable comparisons among 3D structures. The similarity score ranges from 0 to 1, where 1 indicates identical feature-annotated 3D structures.

For most tasks, we used a single atomic feature: the atomic number (Z) of each atom, which encodes elemental identity and complements the 3D geometry. To avoid heavy elements dominating the similarity score, this value was scaled by a square root transformation (√Z), a purely technical adjustment. As discussed elsewhere,^119^ this choice balances chemical specificity and robustness, particularly for metal-containing compounds, where properties such as formal charge or electron count may be ambiguous or inconsistently reported. Two benchmark tasks specifically assess the generation of charged structures and therefore include formal charge as an additional atomic feature in the HSR score. The intrinsic scalability of HSR, its ability to incorporate any number and type of atomic features within the same implementation, makes property-sensitive benchmarks easy to create. Moreover, because HSR is computationally efficient and captures both geometric and atomic information in a general manner, it can be applied to any benchmark task regardless of molecular complexity. Consequently, the difficulty of present or future benchmarks can be tuned without altering the scoring function, simply by selecting targets of the desired complexity and defining a sufficiently dissimilar starting population (see below).

The targets defined in 3DOpt are 3D structures from the CSD, which cannot be redistributed with the benchmark; however, computing the HSR similarity for a pair of 3D structures requires only their HSR fingerprints. For the target molecules, these fingerprints were precomputed and are distributed with the HSR scoring function. This design enables candidate evaluation without access to the original CSD entries and eliminates the need for a CSD license.

To maintain continuity with existing MolScore scoring functions, which primarily operate on SMILES, the HSR implementation also accepts SMILES input alongside 3D structures. When SMILES are provided, a single 3D conformer is automatically generated and used to compute the HSR fingerprint. Users may choose from three conformer generation pipelines: the CSD Python API^120^ (hereafter “CCDC”), Open Babel,^75^ and RDKit.^121^ Each pipeline outputs a single final 3D conformer under its default settings. Importantly, only the CCDC pipeline performs an internal conformational sampling procedure before selecting its final structure, whereas the Open Babel and RDKit pipelines generate a single 3D structure directly from coordinate generation followed by force-field minimization (see Section S3.1.1 of the Supporting Information).

This capability simplifies the integration of SMILES-based generative methods with the HSR scoring function, enabling their outputs to be evaluated in a 3D-aware manner without additional preprocessing.

### Starting Populations

With the targets and scoring function defined, each benchmark task is completed by defining a starting population, that is, the set of prescored structures that serves as the initial pool from which a generative method begins its exploration of new structures.

Because the starting population consists of scored structures, it is specific to each benchmark task and plays a crucial role in determining the practical difficulty of the task. However, because 3D structures from the CSD cannot be distributed, 3DOpt provides the CSD refcodes. Any user or generative method requiring 3D information from the starting population must retrieve these structures directly. For methods that do not use initial 3D structural information, the canonical SMILES declared in the CSD is used to define the starting population.

For each benchmark task, the starting population is constructed by selecting from the viable structures (see the Data Set Generation section, Figure 3) all molecules whose HSR similarity score to the task’s target is below 0.5. This threshold ensures that the starting population excludes molecules highly similar to the target, thereby providing a meaningful challenge for the generative methods. Furthermore, because all viable molecules meeting this criterion are included, the initial sets exhibit substantial structural and score diversity (see Section S2 of the Supporting Information for size and similarity distribution).

**Figure 3:**
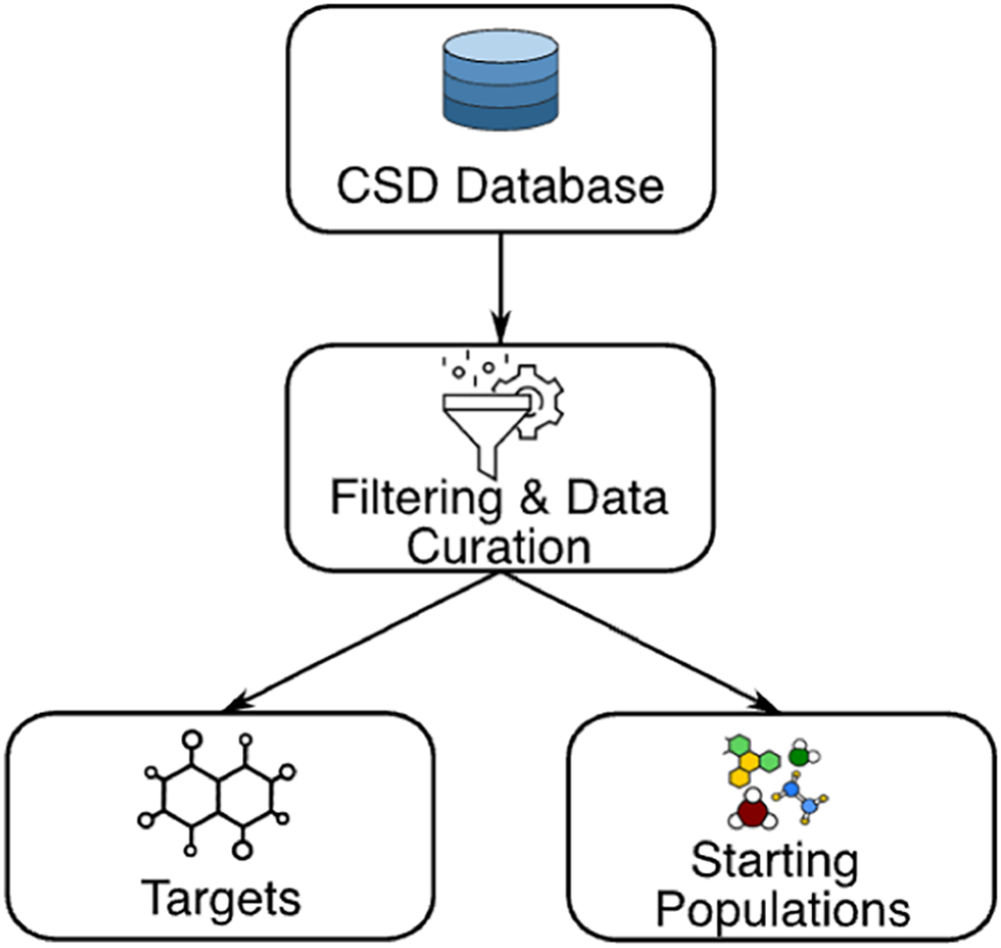
Workflow for selecting target molecules and corresponding starting populations via filtering and data curation of the Cambridge Structural Database (CSD).

To characterize the difficulty of each structural rediscovery task, we employed a coverage metric that quantifies the extent to which the target’s structural information is represented in the corresponding starting population. This metric is based on Connected Atom Environments (CAEs), defined as 3D fragments that include the coordinates, identity, and charge of an atom and all its directly bonded neighbors (as specified in the CSD entry). Unlike the BCPs used to describe target molecules in chemically intuitive terms, CAEs do not account for bond features and are therefore unsuitable for target selection. However, unlike BCPs, CAEs can be extracted and detected automatically, enabling straightforward measurement of how much of a target’s structural information is covered by the corresponding starting population. A coverage value near 100% indicates that all necessary 3D fragments are present, making the challenge primarily one of recombination. Conversely, low coverage values imply that the method must generate novel 3D fragments (CAEs absent from the starting population), thereby increasing task difficulty. Further details on starting population selection, CAE extraction, and coverage analysis are provided in Section S2 of the Supporting Information.

### Baseline Methods

To establish baseline scores for comparison with other generative methods and to demonstrate the functionality of 3DOpt, we implemented and evaluated two baseline approaches: a random sampler and an evolutionary algorithm.

The random sampler serves as a minimal, nonoptimizing reference. For each benchmark task, it samples a fixed number of molecules (100) directly from the starting population. We evaluated this baseline using two strategies for obtaining 3D structures: (i) retrieving experimentally determined structures from the CSD and (ii) generating 3D structures on-the-fly from SMILES representations using one of the SMILES-to-3D pipelines available in the HSR scoring function (CCDC, Open Babel, and RDKit; see Scoring Functions). This SMILES-based setup was designed to assess how inherent differences in conformer generation pipelines influence the performance of 3D generative design methods and how these compare to using experimentally derived CSD structures. It is important to note that only the CCDC pipeline includes an integrated conformational sampling step when generating the 3D structures. As a result, one would expect that adding extensive conformational sampling to the other pipelines could improve their performance. However, to ensure that the baseline results reflect the behavior of general-purpose and minimally tuned pipelines, we intentionally limited the use of conformational sampling and relied on default settings wherever possible (see Section S3.1.1 of the Supporting Information). Our aim was to provide baseline results that represent performance under unoptimized, broadly applicable conditions, rather than results that depend on task-specific parameter tuning.

The other baseline is an evolutionary algorithm adapted from ChemGE.^122^ In ChemGE, SMILES representations are encoded as numerical vectors according to a context-free grammar, enabling the application of standard evolutionary operations, such as mutation and recombination, to generate new molecular structures. The original ChemGE was designed for small organic molecules; we modified and extended its grammar to support SMILES of inorganic compounds (see Section S3.1 of the Supporting Information). Because ChemGE operates exclusively on SMILES representations, 3D structures were generated on-the-fly using one of the three SMILES-to-3D pipelines available in the HSR scoring function. In all ChemGE experiments, the initial population was constructed from the top 100 molecules selected from the starting populations. The algorithm was then run for 100 generations, with 50 molecules mutated at each generation. The performance was evaluated based on the molecules in the final generation.

Overall, each of the seven baseline configurations, defined by combining a baseline method with a 3D structure generation pipeline, was executed in 10 independent runs. All reported performance metrics are averages over these runs.

### Benchmark Metrics

The 3DOpt benchmark metrics assess the performance of a generative method both for individual tasks and across the entire benchmark. For each task *i,* the performance from a single run of the method is measured by the *task score* (*T*_*i*_, eq 1), defined as

1 $$T_{i}=\frac{1}{2}\left(s_{1}+\frac{1}{10}\overset{10}{\underset{j=1}{\sum }}s_{j}\right)$$

In eq 1, *s*_*j*_ denotes the score of the *j*-th top-ranking molecule in a given run. Two design principles underlie this definition. First, the task score is computed from the top ten candidates rather than solely from the best candidate, reducing the impact of occasional outliers and assessing the method’s ability to consistently generate high-quality solutions. However, the weighting scheme strongly favors the best candidate, which contributes 55% of the total task score, thereby ensuring that exceptional solutions receive appropriate emphasis. Second, in the context of 3D structures, duplicates are defined as structures with identical constitution and 3D coordinates within a small tolerance (e.g., 0.001 Å), irrespective of atom ordering or formal connectivity. Consequently, 3DOpt is insensitive to strict connectivity descriptions, mitigating ambiguities in representing unusual bonding patterns. Because generative methods are not expected to implement duplicate filtering under this definition, duplicates are permitted. The theoretical maximum task score is 1.0, achieved by reproducing the target structure in all ten top-ranked candidates.

The overall performance, expressed as the *benchmark score* (*B*), is obtained by summing the task scores across all 32 tasks, producing a single aggregate value in the range 0–32. Higher scores indicate greater ability to systematically generate molecular structures that closely match the predefined targets.

While the benchmark score (*B*) provides a high-level measure of overall performance, an analysis of individual task scores (*T*_*i*_) reveals specific strengths and limitations of a generative method. For example, a method may excel on organic targets but may struggle with inorganic ones or successfully reproduce common structural motifs while failing on more complex patterns. Such task-level insights are essential for guiding targeted improvements in generative algorithms and are not captured by the aggregate 3DOpt benchmark scores.

### MolScore Implementation

The 3DOpt benchmark is implemented within the MolScore framework,^45^ which provides a standardized and extensible platform for running and comparing multiple benchmarks, such as GuacaMol and MolOpt. MolScore includes a broad collection of scoring functions and metrics that can be flexibly combined to define new tasks. It also offers an interface for integrating generative methods, enabling evaluation under diverse setups with minimal overhead. In addition, MolScore supports customization and extension of tasks, facilitating both execution and further development of the 3DOpt benchmark. As part of this work, MolScore has been extended to support molecular representations beyond SMILES, provided they are compatible with the chosen scoring function, and to include an HSR-based scoring function.

## Results and Discussion

The benchmark scores (*B*) for each baseline configuration are summarized in Table 1. The task scores (*T*_*i*_) for the random sampler and ChemGE baselines are shown in Figures 4 and 7, respectively.

**Figure 4:**
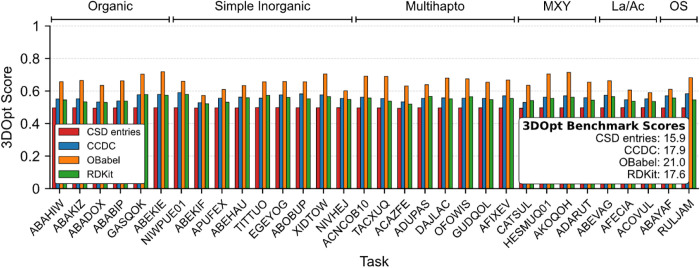
Task scores and benchmark scores for the random sampler baseline method combined with either direct extraction of 3D structures from CSD (“CSD entries”) or SMILES-to-3D conversion (“CCDC”, “OBabel”, or “RDKit”). For graphical clarity, tasks are grouped into categories based on the most significant structural features of their target molecules: “Organic” indicates organic molecules containing only atoms from the *s*- and *p*-blocks; “Simple Inorganic” involves *d*-block atoms in relatively simple topologies; “Multihapto” involves multihapto ligands; “MXY” involves metal-bridging atoms and agostic bonds; “La/Ac” involves lanthanides or actinides; and “OS” involves atoms in an intended, nontrivial, oxidation state. See Section S1.3 of the Supporting Information for more information on the target categories.

**Table 1:** Benchmark Scores (*B*) for Each Combination of Baseline and 3D Generation Approach

benchmark scores (*B*)^a^		
3D generation approach	random sampler	ChemGE
CSD entries	15.89 ± 0.01	N.A.
CCDC	17.90 ± 0.19	18.71 ± 0.01
OBabel	20.98 ± 0.11	20.98 ± 0.05
RDKit	17.64 ± 0.13	18.85 ± 0.08

a Values are reported as mean ± standard deviation over 10 independent runs.

Interpretation of these results requires understanding the starting populations. Each task’s starting population consists of all CSD entries with a maximum similarity score of 0.5 to the task target (see Methods). The distribution of scores in the starting population is therefore truncated and strongly skewed toward 0.5 (see Section S2 of the Supporting Information). Random sampling from such populations cannot yield task score*s* above 0.5 and is highly likely to select candidates with similarity scores close to 0.5. This pattern is observed when the baseline method extracts 3D structures directly from CSD (Figure 4, series *CSD entries*). In this case, the baseline method does not generate any new structures, and the similarity scores are identical to those in the starting population.

By contrast, when 3D structures are generated on-the-fly from the SMILES of the CSD entries selected by the baseline method using a conformer generator, the scores are always, and often substantially, higher than 0.5 (Figure 4). The 3D structures generated by the SMILES-to-3D pipelines may differ from the corresponding CSD structures in both molecular constitution and geometry. As elaborated below, such deviations can arise from limitations in the SMILES representation (e.g., incomplete encoding of coordination environments) as well as from the inability of some pipelines to correctly model certain atom environments, leading to geometric distortions. Consequently, the generated structure for a given CSD entry may be either more or less similar to the target, reflected in higher or lower HSR scores, than the experimental CSD geometry. However, the calculation of the task score considers only the molecules with the highest HSR score and therefore amplifies any coincidental structural distortion that results in an HSR score higher than that of the corresponding CSD structure. Consequently, the task score for any baseline including SMILES-to-3D conversion is typically higher than that computed from the corresponding CSD entries.

The deviation in HSR scores between the CSD structure and SMILES-to-3D generated structure for a given entry may arise from several factors, including limitations of SMILES representation, distortion of challenging geometries, conformational sampling and flexibility leading to large changes in molecular shape among the lowest-energy conformers, and even packing effects in the crystal structure.^123–125^

Notably, the canonical SMILES provided by the CSD and used here as 1D/2D representations do not include explicit hydrogens except for polar ones. Conformer generators typically infer hydrogen counts using valence rules, but for structures that are not well described by these rules—such as some considered here—this process can result in missing hydrogen atoms. Although explicit-hydrogen SMILES can be obtained with tools other than CCDC, several considerations led us to retain the canonical SMILES from the CSD. First, most conformer generators, including all those tested here, have been developed and optimized for implicit-hydrogen SMILES.^126–129^ Indeed, attempts to use explicit-hydrogen SMILES result in substantially worse performance than with canonical SMILES (see Section S3.5 of the Supporting Information).

Moreover, in this work, SMILES are used to run baseline methods, whose role is to establish a lower bound on performance rather than to evaluate fully optimized input representations. More generally, SMILES themselves may not be sufficiently robust or expressive to serve as inputs for generative methods,^85^ even before considering the added complexity of metals and nonvalence bonding motifs.^130^ Optimizing molecular inputs and representations for this purpose is therefore an important but separate challenge, and one that lies beyond the scope of the present work. Finally, the limitations of SMILES are expected to affect all of the SMILES-driven baselines similarly.

Beyond the effect of the input SMILES, the increase in task scores for SMILES-based baselines can be interpreted as a measure of the geometrical distortion introduced by the corresponding SMILES-to-3D conversion. Accordingly, the results in Figure 4 show that the RDKit conformer generator produces the least distortion with scores only slightly above the 0.5 threshold. CCDC gives comparable, yet slightly worse results, while the distortion reflected by higher task scores is most pronounced for the OBabel SMILES-to-3D conversion. Clearly, this ranking depends on the specific chemical space, which is highly populated with challenging organometallic compounds. Nevertheless, the results so far suggest that the CCDC and RDKit generators most accurately reproduce crystal structures. In our experiments, the internal conformational sampling performed by the CCDC pipeline did not materially affect the resulting HSR scores. This suggests that for molecules with inherently low shape similarity to the target, additional conformer sampling offers limited benefit. Consequently, extensive sampling would also be expected to have only a modest impact on the RDKit and Open Babel pipelines, which currently generate a single-minimized 3D structure. Instead, for molecules that are constitutionally closer to the target, such as those that should eventually be expected by any effective 3D generator other than a random sampler, extensive conformational sampling should become beneficial.

Notably, the task score considers only the successfully evaluated molecules, but the success rate (i.e., the fraction of molecules for which any 3D structure was produced regardless of its quality) varies significantly across conformer generators. OBabel achieves the highest success rate (94%), indicating that it rarely fails to generate a 3D structure for a given SMILES. However, as shown by the high task scores discussed above, this robustness comes at the expense of accuracy. The CCDC generator reached a success rate of 79%, although this is partly attributable to the 10-s timeout applied in these experiments. RDKit has the lowest success rate (65%), reflecting its limitations when handling metal-containing molecules that populate the chemical space considered here. Hence, although the task scores from CCDC and RDKit are comparable, the success rate suggests that the CCDC conformer generator is the most suitable for the chemical space considered in the benchmark.

To assess the capabilities and limitations of the conformer generators, we evaluated their ability to reconstruct the 3D structure of each target molecule from its CSD-derived SMILES. This experiment probes the upper performance limits of these methods. Because conformer generation involves stochastic components, repeated SMILES-to-3D conversions can yield different results. Each target was therefore processed by every generator in 10 independent runs with distinct random seeds, and the averages across the runs are reported in Figure 5.

**Figure 5:**
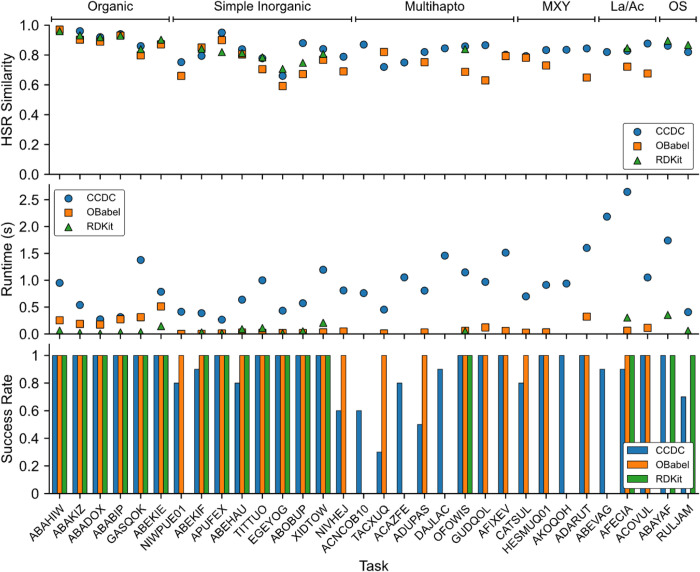
Average success rates, runtimes, and mean HSR similarity for SMILES-to-3D conversion of the benchmark molecules. Values are averaged over 10 independent runs per generator, each starting from the same SMILES representation.

CCDC successfully generated 3D structures for all 32 targets across runs, achieving an average success rate of 89%. Occasional failures occurred for a few challenging molecules when generation exceeded the timeout, and these failures were consistent across runs, indicating that certain structural motifs pose difficulties. This success rate is close to that observed for random sampler runs (79%), suggesting the robustness of CCDC for CSD-derived molecules. In contrast, OBabel and RDKit achieved lower success rates (78% and 53%, respectively) and consistently failed on the same targets. Thus, their success rates reflect the fraction of benchmark molecules each method can handle: OBabel generated 3D structures for 25 of 32 targets, while RDKit managed only 17. The distribution of 0-success rate over the categories clearly shows the struggle of RDKit with multihapto, bridging ligands (i.e., “MXY” category) and lanthanides/actinides compounds.

Figure 5 also summarizes average runtimes and mean HSR similarities for each 3D generator. When considering only the 15 targets for which all methods successfully generated a structure, CCDC and RDKit achieve comparable mean HSR similarities (0.86 and 0.85, respectively), both higher than those of OBabel (0.80). However, when averaging over all 32 benchmark targets and assigning a similarity of zero to failures, CCDC scores 0.84, while OBabel and RDKit drop to 0.60 and 0.45, respectively, reflecting their lower success rates.

The accuracy achieved by CCDC comes at a substantial computational cost: its mean generation time per structure is 0.947 s (with a 10-s timeout), compared to only 0.108 s for OBabel and 0.094 s for RDKit. This difference is primarily due to the additional conformational sampling step performed within the CCDC pipeline. The cost of such sampling, like any overhead required to generate a single 3D structure, should be taken into account when comparing different 3D generators as it directly affects overall efficiency.

In MolScore, sample efficiency^30^ is typically measured in terms of “oracle calls”, i.e., evaluations of the scoring function. However, because conformational sampling occurs before the scoring function is invoked and is independent of it, this cost is not reflected in the oracle-call metric. Furthermore, the diversity of available sampling strategies (e.g., stochastic sampling, systematic searches, or MD-based approaches) makes it impractical to define a universal and consistent constraint on conformer sampling across all 3D generators. For these reasons, cost-based comparisons must rely on direct runtime measurements obtained on consistent hardware, as illustrated in Figure 5 for individual molecules and in the benchmark-wide runtime analyses.

Overall, CCDC delivers the highest accuracy and coverage but at the cost of significantly longer runtimes and occasional failures. OBabel and RDKit are faster but fail to generate valid 3D structures for a substantial fraction of benchmark targets, underscoring inherent limitations when handling structurally complex or nonstandard molecules.

Beyond aggregate performance metrics, an analysis of individual structures reveals additional limitations in the quality and reliability of the 3D geometries produced by the conformer generators. The target with the CSD entry code OFOWIS was selected as an illustrative example because it combines several challenges for the baseline methods: ambiguous SMILES representation, the presence of dative bonds, multihapto interactions involving aromatic systems, and stereogenic centers. Moreover, this molecule exhibits very limited conformational flexibility, meaning that the internal sampling procedure in the CCDC pipeline has a minimal opportunity to improve the generated geometry. Figure 6 shows the original structure, a reference variant where two vinyl hydrogens were removed without geometric relaxation, and the structures generated by CCDC, RDKit, and OBabel from the CSD-derived canonical SMILES representation.

**Figure 6:**
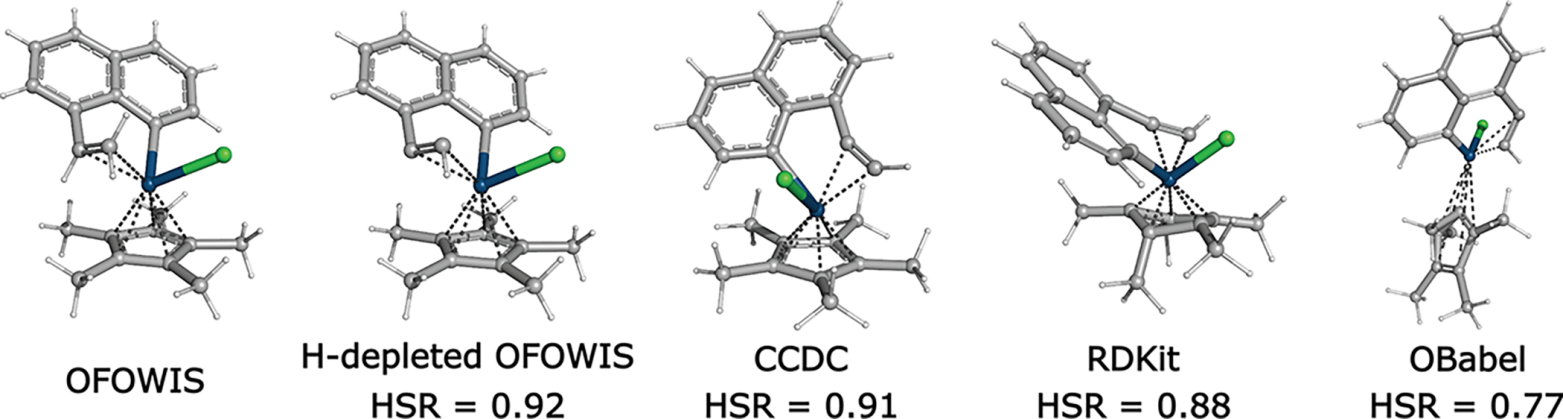
Original OFOWIS structure (CSD entry), an H-depleted variant (two vinyl hydrogens removed without relaxation), and structures generated from the CSD canonical SMILES using CCDC, RDKit, and OBabel. Reported values indicate HSR similarity to the original OFOWIS structure.

The canonical SMILES produced by CSD for this target is ambiguous because it omits two vinyl hydrogens and does not encode the dative and multihapto metal–cyclopentadienyl bonding. To assess the impact of these omissions, we compared the original structure to a variant with the vinyl hydrogen atoms removed. The resulting HSR similarity (0.92–0.93, depending on which terminal hydrogen is retained; 0.92 shown in Figure 6) demonstrates that the loss of these atoms alone substantially reduces the similarity measure.

When provided with this incomplete SMILES representation, all generators produce structures where valence constraints enforce distorted geometries consistent with the SMILES specification. Among them, CCDC achieves the highest similarity (0.91), approaching the upper bound set by hydrogen omission. By contrast, RDKit (0.88) and OBabel (0.77) exhibit additional distortions, primarily involving the metal–cyclopentadienyl system, reflecting inadequate treatment of aromaticity combined with multihapto bonding interactions.

Although CCDC achieves the highest similarity score among the SMILES-to-3D generators (Figure 6), its structure is enantiomeric to that of the target. The HSR similarity method can optionally distinguish enantiomers, provided that they belong to the same types of enantiomerism (e.g., central or axial). This introduces a potential source of inconsistency if chirality discrimination is applied across the chemically unrelated cases. To avoid this, chirality discrimination is disabled in the HSR similarity scoring function used in 3DOpt. For OFOWIS, however, computing chirality-aware similarity reveals shape differences larger than those suggested by the default chirality-unaware values: for CCDC, the HSR similarity drops from 0.91 to 0.82; for RDKit, from 0.88 to 0.81; and for OBabel, from 0.77 to 0.75. While these chirality-aware values highlight differences that are visually evident, we consider this effect to be less critical than retaining consistency across different enantiomerism types. Therefore, we recommend using chirality-unaware shape similarity in the benchmark and, if chirality becomes relevant in future benchmarks, applying chirality-aware similarity only when chirality-unaware similarity exceeds a very high threshold (e.g., > 0.98).

Overall, the OFOWIS example illustrates the typical challenges in generating constitutionally and structurally faithful 3D geometries from low-dimensional representations of complex molecules, particularly when key bonding patterns are not captured by the representation itself or by the valence rules underlying representations, such as SMILES. Despite these limitations, many generative methods rely on stringlike chemical representations and delegate the conversion to 3D structures to downstream postdesign processing. This approach is appealing because design algorithms that manipulate string-based representations are straightforward to implement and computationally efficient. For this reason, we adopted a string-based representation for the baseline methods: it reflects a simple design strategy that is common among current generative methods that are not explicitly intended to produce 3D structures.

In summary, the results obtained by random sampling with any of the conformer generators represent the lower performance limits of generative methods (Table 1). The benchmark scores (*B*) are at least 10 units lower than the theoretical maximum (i.e., 32), leaving room for evaluating methods that include an actual design rather than random sampling. Notably, the 5-unit range in the benchmark scores of the random sampler indicates that geometric distortion can substantially affect the perceived performance. While it is relatively easy to increase HSR similarity from 0.5 to 0.7 through geometric distortion alone, achieving high HSR similarity (e.g., > 0.95) requires both constitutional and geometric consistency with the target molecules, highlighting the nonlinear nature of the benchmark scoring scale.^119^

Unlike the random sampler, the second baseline uses an evolutionary algorithm to optimize candidate structures toward a given goal. It employs a customized version of ChemGE^122^ adapted to handle both organic and inorganic molecules. SMILES are converted to 3D structures on-the-fly using the HSR scoring pipelines (CCDC, Open Babel, and RDKit). No further tuning was applied; this implementation remained a baseline.

ChemGE achieves higher overall benchmark scores than the random sampler for CCDC and RDKit, while OBabel’s score remains unchanged (Table 1). At the task level (Figures 7 and 8), OBabel shows an approximately even split between improvements and declines when moving from random sampling to ChemGE. In contrast, CCDC exhibits only four tasks where ChemGE performs slightly worse, whereas RDKit shows consistent improvement for every target. A closer look reveals that these results are linked to the success rates of conformer generators processing SMILES generated by ChemGE, which, although syntactically valid, can correspond to chemically implausible molecules. Compared to the random sampler, success rates drop sharply for CCDC (79% to 45%) and OBabel (94% to 58%), but only slightly for RDKit (65% to 63%).

**Figure 7:**
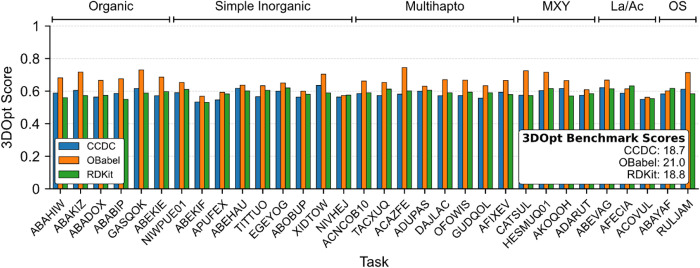
Task scores and benchmark scores for the ChemGE baseline method coupled with SMILES-to-3D conversion using CCDC, OBabel, or RDKit.

**Figure 8:**
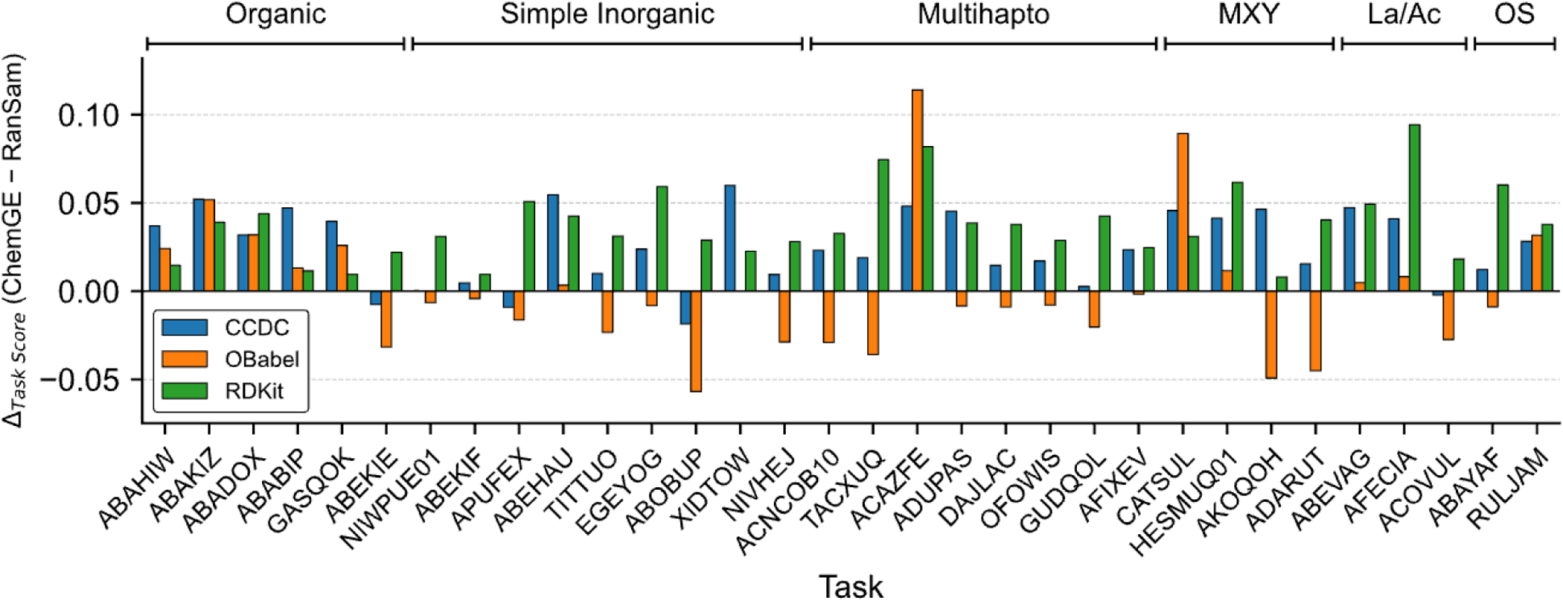
Differences in task scores between the ChemGE and Random Sampler baselines for each SMILES-to-3D conversion method. Positive values indicate higher ChemGE performance.

This suggests that RDKit is considerably more robust in generating 3D structures from the mutated SMILES produced by ChemGE. Remarkably, RDKit moves from being the least successful method with the random sampler to being the most successful under ChemGE. Such robustness likely contributes to its consistently higher task scores, as a greater fraction of optimized SMILES survive evaluation.

In contrast to RDKit’s robustness, CCDC and especially OBabel suffer a substantial reduction in success rate under ChemGE, meaning that fewer optimized SMILES can be converted to 3D structures. This severely limits the chance of producing high-scoring molecules by design. In effect, the difficulty of converting a low-dimensional representation into a valid 3D structure constrains the design capability of the modified ChemGE algorithm. This outcome is not unexpected, as it underscores the motivation for developing generative methods that operate directly in 3D.^48^

Overall, even when some baseline methods achieve relatively high task scores, these values remain well below the upper limit of the similarity metrics (1.0). Moreover, because the task score is an aggregate measure (as detailed in the Methods section), only values approaching 1 can be considered strong evidence of systematic and successful 3D structure rediscovery. In fact, with a maximum benchmark score (*B*) of ∼21, the SMILES-based baselines establish a relatively low starting point, leaving ample room on the scale for evaluating improved 3D generative methods, whether these rely on 1D/2D molecular representations or operate directly in 3D space.

Beyond SMILES-based approaches, generative methods that directly generate and optimize molecular structures in three dimensions (hereafter referred to as direct methods) may ultimately provide highly effective solutions to the challenges posed by 3DOpt. However, no such direct method is included here as a baseline because, at present, none simultaneously satisfies the requirements for a general-purpose evaluation, namely, a redistributable implementation, applicability across both organic and inorganic chemistry, and independence from external structural context. This reflects the current state of the field rather than a limitation of the benchmark. Accordingly, 3DOpt is intentionally designed as an open and extensible framework to motivate and fairly evaluate emerging 3D generative methods as they become available; this anticipatory benchmark design mirrors the virtuous cycle observed in other fields,^21,24–26^ where the early establishment of shared benchmarks helped orient research efforts and stimulate methodological development. Developers of emerging 3D generative frameworks are explicitly invited to integrate their methods via MolScore.

Most of the currently available direct 3D generative methods are tailored to specific contexts. In drug design, for example, 3D-based approaches often rely on structure-based design strategies, where a binding pocket, a pharmacophore, or anchor fragments must be provided.^131–135^ Such specialized methods can be evaluated within dedicated benchmarks for structure-based molecular generation.^32,33^ However, they are incompatible with general-purpose 3D structure generation and cannot handle inorganic compounds because of restrictions on permitted atom types.^134,135^

In contrast to structure-based approaches, ligand-based methods generate or optimize molecules directly in 3D without requiring pocket information.^136–138^ While this avoids dependence on structural context, these approaches are still developed exclusively for organic, drug-like molecules and are not applicable to the inorganic compounds included in 3DOpt.

Similar constraints exclude certain direct 3D generative methods that can design inorganic structures but are limited to highly specific systems, such as octahedral-only organometallic complexes^103^ or metal-only and metal oxide clusters.^139^ The broad structural and chemical diversity required by 3DOpt exceeds the capabilities of such methods.

Among direct 3D generative methods independent of pretrained machine learning (ML) models, NaviCatGA^89^ and DENOPTIM^102^ appear to be the most promising candidates. Unfortunately, neither is directly applicable to 3DOpt in its out-of-the-box form, albeit for different reasons.

NaviCatGA^89^ is a genetic algorithm originally developed for homogeneous catalyst design and supports multiple molecular representations, including SMILES, SELFIES, and XYZ coordinates. While NaviCatGA has been applied to both organic and organometallic catalysts,^89,140^ its 3D (XYZ) workflow has so far only been demonstrated on organic systems. In its current implementation, the XYZ workflow is unsuitable as a 3DOpt baseline for several reasons. First, it requires pregenerated fragments and restricts anchor points for assembly to hydrogen positions, substantially limiting connectivity diversity without manually defining metal-containing fragments. Second, the genetic operations are tailored to organic chemistry and are not directly transferable to the inorganic space; for example, recombination (crossover) is restricted to attaching fragments at hydrogen atom positions, and mutations follow organic valence-based rules that do not capture inorganic connectivity. Third, neither the genetic operations nor the local geometry minimization procedure (applied after fragment connection and based on a torsional Lennard–Jones potential) can handle cyclic structures. As a result, multidentate ligands or other cycles like those present in the 3DOpt benchmark cannot be designed or altered. In summary, NaviCatGA cannot be applied without significant changes to its core logic, which are beyond the scope of the present work.

DENOPTIM^102^ offers greater flexibility by allowing customization of cutting rules that define fragments and attachment points (anchor points), as well as connectivity rules, enabling deployment across both organic and inorganic systems. Its genetic operators also support cyclic structures, overcoming a key limitation of NaviCatGA.^141^ However, DENOPTIM’s 3D geometry refinement and ring handling rely on a customized version of Tinker^142^ that implements a tailored ring-closure potential.^141^ Because Tinker’s license prohibits redistribution of modified versions, DENOPTIM cannot be provided as a fully reproducible baseline within 3DOpt, restricting its use for benchmarking purposes.

Beyond rule-based approaches like NaviCatGA and DENOPTIM, several direct 3D generative methods leverage pretrained machine learning models.^143–147^ While highly promising, these models are typically trained on narrow chemical domains, such as small drug-like organics, and are not intended for broad application beyond their original scope. Applying them to 3DOpt would therefore require large-scale retraining on data sets with greater structural diversity, such as large portions of the CSD. Importantly, retraining directly on the benchmark targets would introduce bias, making the generator nontransferable to other tasks. Retraining on the initial populations or their union is feasible. However, this constitutes the development of a new, generalized 3D generative method rather than the presentation of the 3DOpt benchmark.

Given these limitations, no direct 3D generative method was included as a baseline in this work. The hypothetical application of any currently available task-specific 3D generative method would lead to failures across many of the benchmark tasks, preventing it from serving as a meaningful baseline whose role is to provide broadly applicable reference results for comparing new methods. Instead, the SMILES-driven baseline results presented here provide a conservative benchmark. They highlight that meaningful progress will require either a shift toward direct 3D-capable molecular representations or significant advances in lower-dimensional representations and conformer generation to handle a wider spectrum of chemical structures robustly, accurately, and efficiently.

The 3DOpt benchmark itself also faces a key limitation: its inability to assess the chemical validity of generated structures. In existing benchmarks other than 3DOpt,^18,27,45,30^ *validity* usually refers to whether a molecular representation is syntactically correct and encodes a theoretically possible molecule. Typical checks include verifying the syntax of strings such as SMILES or SELFIES and confirming that a representation can be parsed by cheminformatics toolkits, such as RDKit.^18,27^

In the context of 3D molecular generation, however, validity requires a definition that is independent of the formal constraints of lower-dimensional representations. Theoretically, one could define a valid structure (neutral or charged molecule)^148^ as any configuration corresponding to a minimum on a potential energy surface deep enough to host a vibrational mode. In principle, such assessments could be performed using fast quantum chemical methods such as xTB^149^ or even DFT, but these remain too computationally expensive for large-scale benchmarking. Machine-learning potentials^150^ offer an appealing alternative by providing rapid energy estimates. However, current models are primarily trained on organic, drug-like molecules,^151–153^ and although promising efforts aim to extend them to transition-metal complexes and broader regions of the periodic table,^154,155^ these approaches are not yet mature enough for routine use and thus cannot serve as a validity check for 3DOpt.

Although implementing ML-based validity checks lies beyond the scope of the present work, such tools represent a natural complement to the strategies discussed above. By identifying molecules with unphysical distortions or chemically implausible environments, they could help prevent artificially inflated benchmark scores, including those occasionally observed for the SMILES-to-3D conformer generators examined here. To enable this evolution, 3DOpt was developed within the MolScore framework, which explicitly supports modular extensions. Users can readily incorporate new validity assessment methods or other custom criteria, ensuring that the benchmark remains adaptable to future methodological advances. In summary, this work underscores key directions for advancing 3D molecular design, including the development of general-purpose 3D generative methods, more robust conformer generation strategies, and reliable tools for assessing chemical validity, especially for chemical spaces beyond traditional organic compounds. By providing a systematic and extensible framework, 3DOpt enables rigorous evaluation of these challenges across a chemically diverse and demanding set of targets. We anticipate that 3DOpt will serve as a foundation for ongoing method development and objective algorithm comparison, ultimately accelerating innovation as generative modeling expands to encompass the full breadth and complexity of chemical spaces.

## Conclusions

The field of 3D molecular generative modeling is advancing rapidly, driven by growing interest in machine-learning approaches for applications in drug discovery, catalysis, and materials science. While several benchmarks exist for de novo design of small organic molecules, none provide a means to evaluate generative methods on 3D structures beyond the drug-like domain.

3DOpt addresses this gap by introducing a framework explicitly designed to test generative methods across both organic and inorganic chemistry. Through rediscovery tasks with selected targets, curated starting populations, and a 3D-aware scoring function based on the hypershape recognition (HSR) similarity method, 3DOpt establishes the first benchmark for general-purpose 3D molecular design across the periodic table.

Baseline results reveal that methods relying on 1D/2D representations and consequently on conformer generators face significant challenges in producing accurate 3D structures for inorganic and structurally complex targets. This underscores the need for improved conformer generation or the development of generative methods that operate directly in 3D.

3DOpt represents a foundational step toward the systematic performance assessment of generative methods for 3D molecular design. By implementing the benchmark within the MolScore platform, we aim to foster continued development of both the benchmark and 3D generative methodologies, ultimately accelerating progress toward robust, general-purpose solutions for the full breadth of the chemical space.

## Supplementary Material



## Data Availability

3DOpt is implemented as a new benchmark within the MolScore framework and is available in the beta version of the forthcoming v2 release of MolScore (https://github.com/MorganCThomas/MolScore/tree/v2.0.0-beta). All baseline experiments reported in this study were conducted using the MolScore baselines repository (https://github.com/MorganCThomas/MolScore_baselines), which provides standardized tools for evaluating generative algorithms across MolScore benchmarks. Materials related to the development of 3DOpt, including selected analyses, figures, and intermediate results presented in this work, are available in the dedicated 3DOpt repository at https://github.com/marcellocostamagna/3DOpt. The complete archive of baseline results generated for this study is publicly available via Zenodo (DOI: 10.5281/zenodo.16602641). This archive contains all run directories and data files for every method and generator evaluated, enabling full reproduction of the reported results.

## Notes

Detailed description of target selection, starting populations, and coverage analysis, along with all computational details and data used and produced in the development of the 3DOpt benchmark (PDF)

## #

Department of Medicine and Center for Biotechnology, College of Medicine and Health Sciences, Khalifa University of Science and Technology, Shakhbout Bin Sultan St., Abu Dhabi, United Arab Emirates
