# Supplementary material for: 3DOpt: Benchmark for Automated Design of 3D Molecular Structures across the Periodic Table
Source: J Chem Inf Model. 2026 May 19;66(11):6387–402. doi: 10.1021/acs.jcim.6c00259 (PMC13250916; doi:10.1021/acs.jcim.6c00259)
Supplement: Supplementary file 1 [file ci6c00259_si_001.pdf]

Supporting Information for

3DOpt: Benchmark for Automated Design of 3D Molecular Structures Across the Periodic Table

Marcello Costamagna,[a] Morgan Thomas,[b,†] Marco Foscato,[,a] Vidar R. Jensen[*,a]*

[a]Department of Chemistry, University of Bergen, Allégaten 41, N-5007, Bergen, Norway

[b]Computational Science Laboratory, Universitat Pompeu Fabra, PRBB, Barcelona, Spain

†Present Address: College of Medicine and Health Sciences, Khalifa University of Science and Technology, Shakhbout Bin Sultan St, Abu Dhabi, United Arab Emirates.

Table of Contents

S.1. SELECTION OF TARGETS S2

S.1.1. Overview and Rationale S2
S.1.2. Basic Connectivity Patterns S2
S.1.2.1. Examples of BCP Detection S4
S.1.3. Targets S5

S.2. STARTING POPULATIONS AND COVERAGE ANALYSIS S17

S.3. COMPUTATIONAL DETAILS S21

S.3.1. Baseline Methods S22
S.3.2. Data Set Generation S22
S.3.3. Targets Selection S22
S.3.4. Starting Populations and Coverage Analysis S23
S.3.5. Generators analysis S23
S.3.5.1. Explicit-hydrogen SMILES analysis S23

S.4. COMPUTATIONAL DATA S23

S.4.1. Baselines Results per Run S23
S.4.1.1. Random Sampler from CCDC entries S23
S.4.1.2. Random Sampler with CCDC S24
S.4.1.3. Random Sampler with RDKit S25
S.4.1.4. Random Sampler with OBabel S26
S.4.1.5. ChemGE with CCDC S27
S.4.1.6. ChemGE with RDKit S28
S.4.1.7. ChemGE with OBabel S29

S.5. REFERENCES S30

S.1. Selection of Targets

S.1.1. Overview and Rationale

The target molecules considered in this work are monomolecular systems. This choice is motivated by the desire to limit the computational cost associated with generating 3D molecular structures. Importantly, this restriction is not inherent to our 3D benchmarking approach based on HSR similarity. Because HSR similarity does not depend on molecular connectivity, it can be applied to any cloud of annotated points in three-dimensional space. In this framework, atoms are treated simply as annotated 3D points, independent of the chemical nature of the system. Consequently, we anticipate future extensions of the method to supramolecular targets—including contact ion pairs, host–guest complexes, and molecular clusters—designed to challenge specialized generators in combination with cost-effective 3D modeling strategies suited for such systems. Nonetheless, the present study focuses exclusively on monomolecular targets.

The selection of the monomolecular target molecules aimed to create a small sample of experimentally determined 3D structures that explore combinations of Basic Connectivity Patterns (BCPs), defined in Section S.1.2, and which represent intuitive molecular orbital-based building blocks for molecular topologies. Notably, rather than opting to reproduce the statistical distribution of such building blocks in known molecules, which would have resulted in a set dominated by undemanding bonding patterns typical of standard organic chemistry (e.g., Csp^3–H, Csp^3–Csp^3, Csp^2=Csp^2), we ensured ample representation of all the eight BCPs to achieve a set of target molecules reflecting the challenges associated with manipulation and design of 3D structures of inorganic and organometallic compounds.

S.1.2. Basic Connectivity Patterns

Basic Connectivity Patterns (BCPs) represent archetypal Connectivity Patterns (CPs) observed in monomolecular systems where atoms are linked via σ- and π-bond components. δ bonds are excluded from consideration because they occur only rarely. CPs represent simplified and intuitive models for how atoms are bonded together to form molecular topologies. More specifically, a CP is an element-independent pair or triad of atoms connected by a formal bond component that can be intuitively described by a localized molecular orbital (MO), such as a Natural Bond Orbital (NBO).[1,2] Importantly, however, CPs are assigned solely on the basis of the chemical the structure, not on analysis of the molecular wavefunction. A CP expresses the most geometry-determining features of a bond component (symmetry, delocalization, cyclicity) and remains element independent. The element independence is essential to limit the number of BCPs to a set small enough to allow for classification via visual inspection of molecular structures. This classification is based on the following criteria:

- **Delocalization**: 2-center or 3-center BCP depending on the number of atoms principally involved in sharing the bonding electrons. If more than two atoms are involved, a third atom is included to define a localized bonding MO that can represent the CP. Larger delocalized systems are divided into overlapping diatomic/triatomic units. Notably, a triatomic CP may involve delocalized or multi-center interactions, e.g., three-center two-electron (*3c–2e*) bonds, multihapto coordination, and bridging coordination.[3,4]
- **Symmetry:** σ or π depending on the symmetry elements of the localized bonding MO of the atom pair/triad, namely the bond axis (diatomic BCP) or

the plane of the triad or a bisecting plane passing through an atom and the opposite bond (triatomic BCP).

- **Cyclicity:** acyclic or cyclic depending on whether the CP is embedded in a cycle of connected atoms involving atoms outside of the CP.

The combination of these criteria defines a set of eight BCPs (Figure S1). These eight BCPs were used to choose target molecules covering all the BCPs in various molecular contexts and to manually classify the CPs of molecules in the current work. Sample classifications are described in the next subsection.

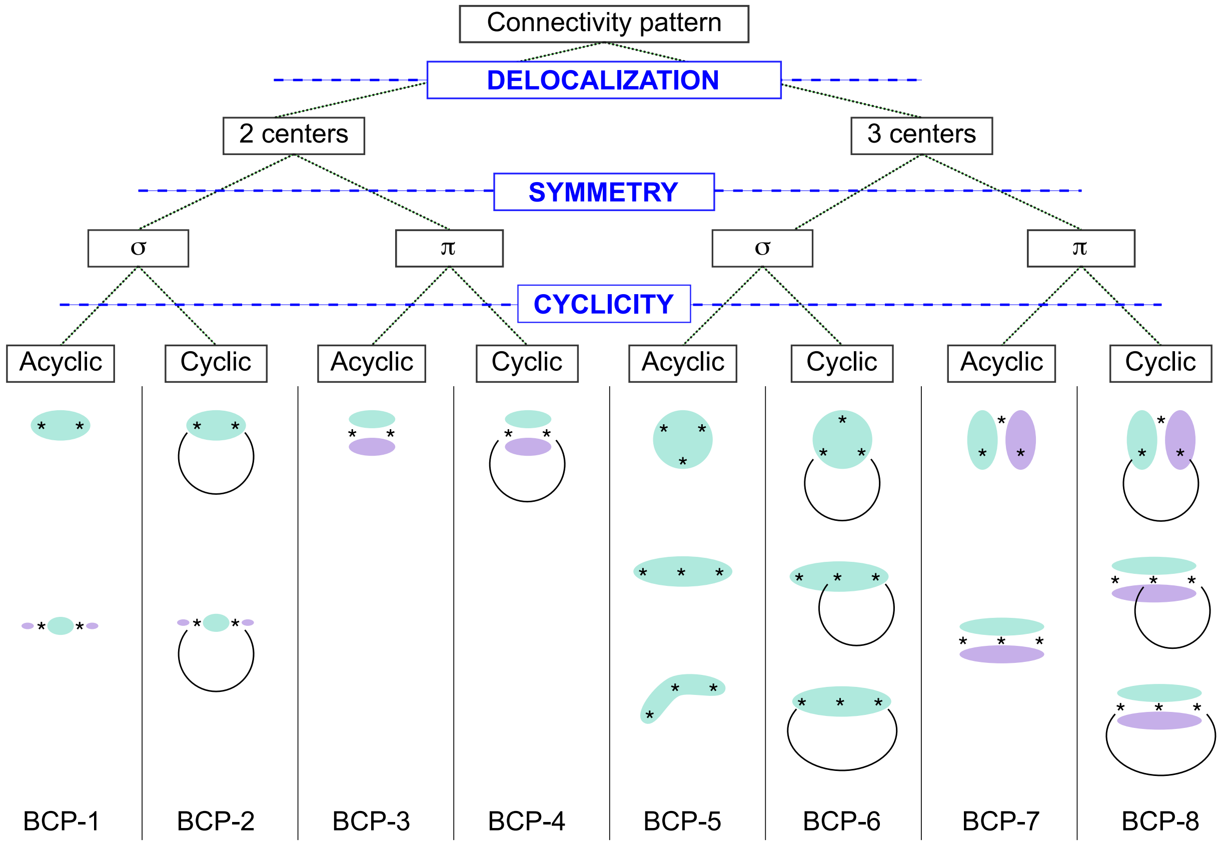

Figure S1. Framework for classifying any Connectivity Pattern (CP) as one of eight Basic Connectivity Patterns (BCPs). The three classification criteria consider the electron delocalization (over two or three atoms), the symmetry (σ or π) of the most representative localized bonding orbital, and the cyclicity (whether the CP is embedded in a cycle). Each BCP is identified by a unique string (i.e., BCP-#) defined at the bottom of the figure, and provided with a non-exhaustive set of schematic examples of the localized MO. Green and violet shapes represent localized MOs with different phases, asterisks represent atoms of any element, and circular arcs represent any cyclic connectivity external to the BCP. The schemes for BCP-1 and BCP-2, exemplify localized orbitals with and without nodal surfaces passing through the atoms to illustrate that such nodes do not affect the CP classification and are, therefore, omitted for the rest of the BCPs. Similarly, the example for BCP-5 shows that the geometrical arrangement (trigonal, linear, or bent) has no effect on the classification as long as the appropriate symmetry elements are considered (i.e., rotation axis for linear, or reflection plane for bent or trigonal triads).

S.1.2.1. Sample BCP Classifications

This section offers two examples that illustrate how the above classification framework is used to identify the BCPs defined in Figure S1 in two non-trivial 3D structures.

The first example considers diborane (B_2H_6), a textbook example of a compound with three-center two-electron bonds. The CSD crystal structure (Refcode: GAFLAA,[5] Figure S2A) shows two hydrogen atoms that form bridges between the boron atoms. The topology of this system is usually best represented by defining connections, i.e., neighboring relations, between each boron and each bridging hydrogen atom (Figure S2B). Notably, this implies that the neighboring relations in the graph representation (Figure S2C) do not represent the usual electron pairs. Yet, this representation offers two kinds of CPs from which we can start the classification process: the terminal B−H pattern, and the internal (bridging) counterpart.

The terminal B−H is described well by a localized (two-center), acyclic, and σ-symmetric bond component. According to Figure S1, these features correspond to BCP-1, i.e., the BCP describing any *2c−2e* σ-bond component, which is thus matched by each of the four terminal B−H CPs of diborane.

In contrast, the internal B−H pattern cannot be represented by a simple diatomic CP because too few electrons are available to populate the hypothetical set of four diatomic BCPs. The internal B−H pattern thus forms part of the *3c−2e* bonds. In other words, the B−H pair must be extended to include another boron atom to describe the connectivity with localized molecular orbitals compatible with the number of electrons: 12 valence electrons in total, with 8 already used by terminal B−H connections, and 4 left for constructing the 4 internal B−H bonds. Conceptually, we can represent the *3c−2e* bond in a simplified manner, with each B−H bond in the resulting B−H−B pattern acting as a 2-electron donor for the other boron atom (Figure S2D). This interaction does not involve any major orbital components of π-symmetry with respect to the plane defined by the three atoms, but the two B−H−B CPs share B atoms and thus form a cycle that makes these CPs cyclic. As a result, the B−H−B connectivity patterns are classified as delocalized, σ-symmetric, and cyclic, thus matching BCP-6.

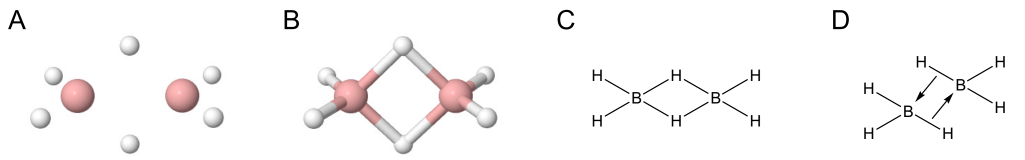

Figure S2. (A) Crystal structure of diborane (CSD Refcode: GAFLAA).[5] White: hydrogen; pink: boron. (B) Crystal structure decorated with connectivity information. (C) Graph representation of diborane's connectivity. (D) Graph representation with the three-center two-electron bonds depicted as *2c−2e* B−H σ-bonds donating their electron pair to the other boron atom.

The second example considers ferrocene ($C_{10}H_{10}Fe$, Figure S3A), a textbook example of a compound involving multihapto bonding. Although there is no consensus on how to define the connectivity of this compound in a graph representation, we chose to adhere to the common practice of defining a connection between each C atom and the Fe atom (Figure S3B). The resulting graph (Figure S3C) shows three types of CPs: C−H, C−C, and Fe−C. The C−H connections are classified as localized σ bonds not embedded in any ring, and thus match BCP-1. Next, the interplay between the C−C and Fe−C connection needs to be considered.

The σ component of the C=C bond can be classified as a single cyclic bond (BCP-2). In contrast, according to the Dewar-Chatt-Duncanson model,[6,7] the π component of each C=C bond donates electron density to the Fe atom and this donation can be interpreted as a form of delocalization, thus justifying the inclusion of the Fe atom into triatomic connectivity patterns with each pair of directly connected C atoms. Within each such triatomic CP, the donation from the π component of the C=C bond to the metal can be described by a three-center localized bonding orbital with σ-symmetry with respect to the C−Fe−C plane. Moreover, this CP is cyclic because of the external connections between each C and Fe. Thus, BCP-6 is a match for each η^2(C=C)Fe unit. Notably, since this interaction consists exclusively of the two electrons from the π component of the C=C bond, the overall η^2(C=C)−Fe interaction can be described as a *3c−2e* bond.[4] Thus, just as diborane, ferrocene involves BCP-6. The Dewar-Chatt-Duncanson model[6,7] also includes back-donation from the metal into the π* of the C=C bond. This component can also be classified as a triatomic BCP, but with π symmetry because of the nodal plane bisecting the C-Fe-C angle. Hence, the back-donation component can be classified as BCP-8: delocalized (metal electrons delocalized over two carbon atoms), π-symmetric, and cyclic. The role of this component, however, must be minor in any η^2(C=C)M system that is not better represented as metallacyclopropane. Conversely, if back-donation were strongly geometry-determining, it would make the metallacyclopropane model be a better fit than the η^2(C=C)M model and, accordingly, the BCP classification would consider each of the three connections in the metallacyclopropane ring as belonging to BCP-2, i.e., single cyclic bonds.

Finally, the symmetry of the η^5 system implies that any triad consisting of Fe and any pair of consecutive C atoms does match BCP-6 and BCP-8, so each η^5-cyclopentadienyl-Fe unit matches both BCP-6 and BCP-8 five times.

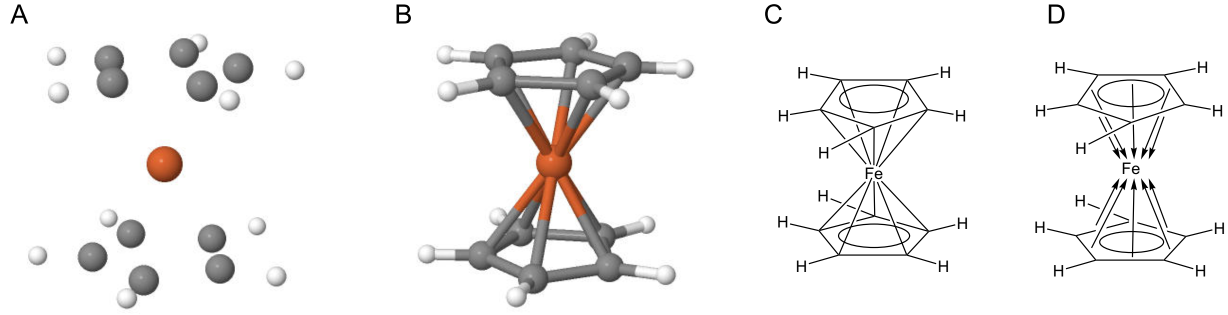

Figure S3. (A) Crystal structure of ferrocene (CSD Refcode: FEROCE04).[8] White: hydrogen; grey: carbon; brown: iron. (B) Crystal structure decorated with connectivity information. (C) Graph representation of ferrocene's connectivity. (D) Graph representation with η^2(C=C)−Fe bonds represented by donation arrows.

S.1.3. Targets

The Basic Connectivity Patterns (BCPs) defined above were used to guide and aid the selection of target molecules. In the following we present the analysis of the targets in terms of BCPs and we also include information as to whether targets match atom arrangements more complex than BCPs, below referred to as *compound patterns*, that are highlighted here because of their chemical relevance.

The chosen target molecules are listed in Table S1 together with their CSD entry code, and 2D representation. For each target, we indicate the matched BCPs, with detailed identification of the molecular fragment matching non-trivial BCPs, as well as any notable compound patterns, and the coordination geometry of metal centers (if any).

To facilitate the navigation of the list of targets and the interpretation of the baseline results, targets are grouped into the following categories based on the most relevant kind of structural features that may challenge molecular structure generators. However, targets were chosen as described in Section S.1.1, so the targets do not intend to sample the diversity within each of the categories. The categories are:

- **Organic**: target molecules containing only atoms from the *s*- and *p*-blocks.
- **Simple Inorganic**: targets containing only one *d*-block atom in relatively simple topologies that still includes ligands ranging from monoatomic, monodentate to polyatomic, polydentate.
- **Multihapto**: targets containing one or more multihapto ligands.
- **MXY**: for targets involving metal-bridging atoms that challenge valence rules, like metal-to-metal bridging atoms and hydrogen atoms of agostic interactions.
- **La/Ac**: for targets containing lanthanides or actinides that can have high and variable coordination number.
- **OS**: for targets involving atoms in an oxidation state that cannot be guessed from the coordinating environment but needs to be specified explicitly.

In addition to the detailed list in Table S1, Figure S4 and Figure S5 summarize the distribution of metal elements and metal coordination geometries present in the list of targets.

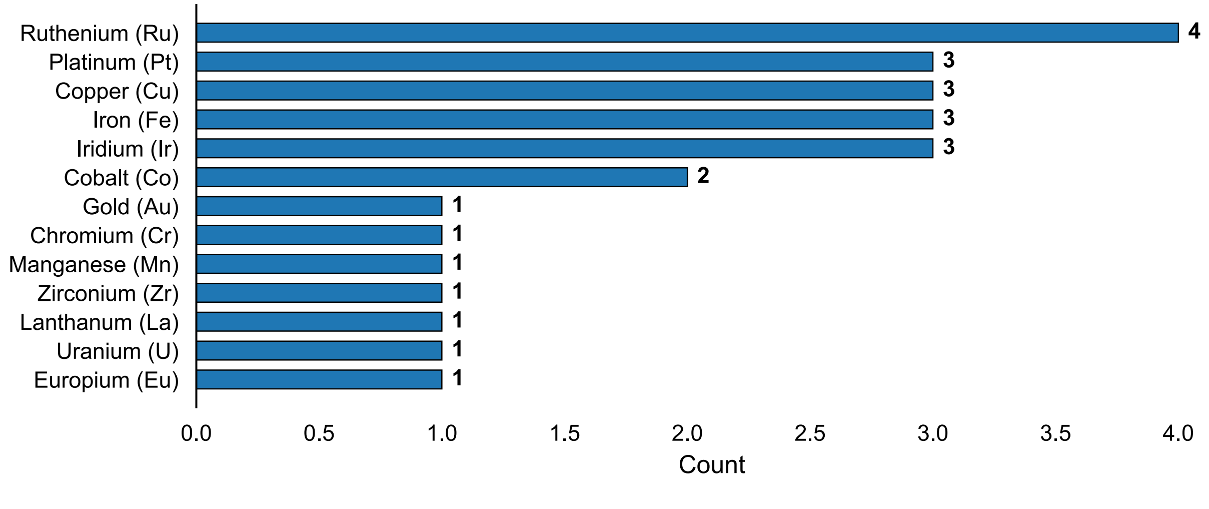

Figure S4. Distribution of metal elements among the inorganic targets.

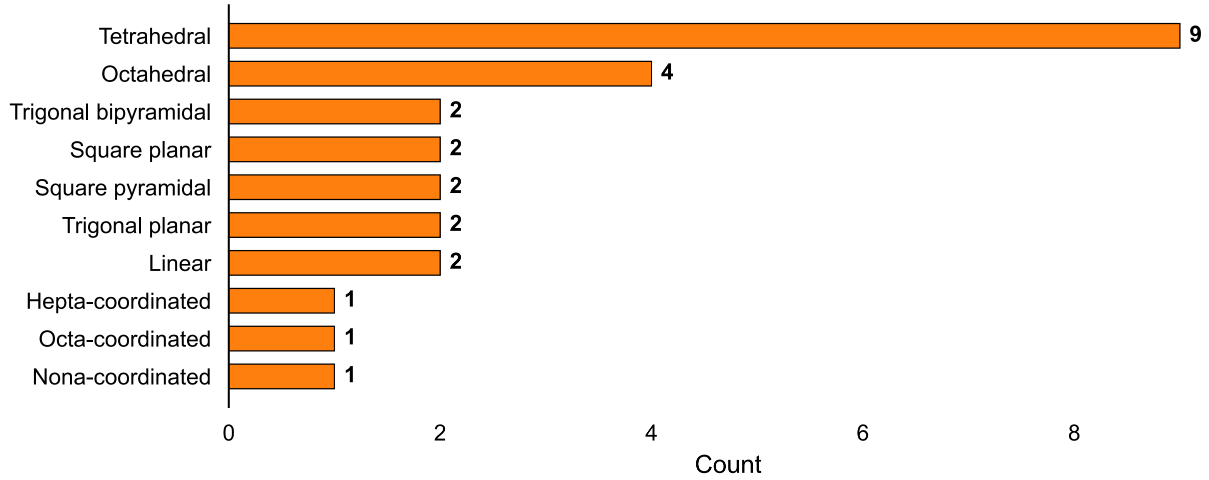

Figure S5. Distribution of metal coordination geometries among the inorganic targets.

Table S1. List of target molecules.

Category: Organic			
**2D Structure,[a] CSD Refcode, and Commented Examples of BCP Matches**	**BCP Matches**	**Compound Patterns**	**Metal Geometry**
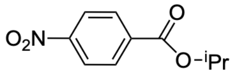<br>ABAHIW<br>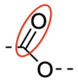 BCP-1: The C=O has a σ bond component matching BCP-1 and a π bond component matching BCP-3. The latter is not highlighted here for clarity.<br>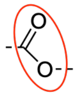 BCP-7: The partial double-bond character of the C−O bond is reflected by the delocalization of its lone pair over the three-center BCP.<br>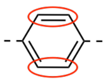 BCP-2: Each aromatic C−C bond has a σ bond component matching BCP-2. For clarity, only two matches are shown.<br>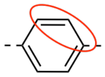 BCP-8: The delocalization of the electrons in the *p*-orbitals is accounted for by 3-center, π symmetry, cyclic BCP.	☒ BCP-1<br>☒ BCP-2<br>☒ BCP-3<br>☐ BCP-4<br>☐ BCP-5<br>☐ BCP-6<br>☒ BCP-7<br>☒ BCP-8	N.A.	N.A.
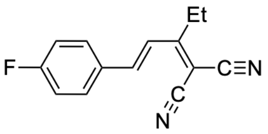<br>ABAKIZ<br>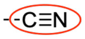 BCP-3: Both π bond components of the triple bond match BCP-3.	☒ BCP-1<br>☒ BCP-2<br>☒ BCP-3<br>☐ BCP-4<br>☐ BCP-5<br>☐ BCP-6<br>☒ BCP-7<br>☒ BCP-8	N.A.	N.A.

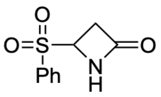<br>ABADOX<br>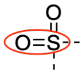 BCP-3: The delocalization of the S=O π bond components is considered negligible because of unfavorable alignment with other π systems.<br>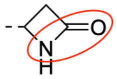 BCP-8: The delocalization of over the C−N bond causes planarity of amide groups and is accounted for by three-center, π-symmetry BCP-7, for acyclic, or BCP-8, for cyclic.	☒ BCP-1<br>☒ BCP-2<br>☒ BCP-3<br>☐ BCP-4<br>☐ BCP-5<br>☐ BCP-6<br>☐ BCP-7<br>☒ BCP-8	N.A.	N.A.
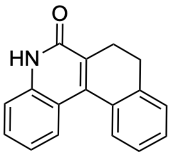<br>ABABIP<br>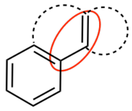 BCP-8: The C=C is part of an extended π system including the aromatic and the amide group. Therefore, it matches BCP-8 instead of BCP-3.	☒ BCP-1<br>☒ BCP-2<br>☐ BCP-3<br>☐ BCP-4<br>☐ BCP-5<br>☐ BCP-6<br>☐ BCP-7<br>☒ BCP-8	Fused rings (aromatic and unsaturated)	N.A.
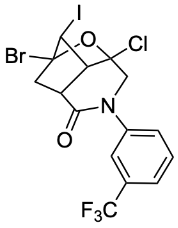<br>GASQOK	☒ BCP-1<br>☒ BCP-2<br>☐ BCP-3<br>☐ BCP-4<br>☐ BCP-5<br>☐ BCP-6<br>☒ BCP-7<br>☒ BCP-8	Fused rings (saturated) Bridged bicyclic rings (saturated)	N.A.
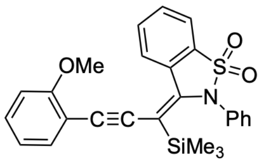<br>ABEKIE	☒ BCP-1<br>☒ BCP-2<br>☒ BCP-3<br>☐ BCP-4<br>☐ BCP-5<br>☐ BCP-6<br>☒ BCP-7<br>☒ BCP-8	Fused rings (aromatic & saturated)	N.A.

Category: Simple Inorganic			
**2D Structure,[a]<br>CSD Refcode,<br>and Commented Examples of BCP Matches**	**BCP Matches**	**Compound Patterns**	**Metal Geometry**
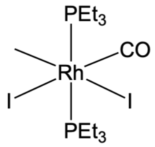<br>NIWPUE01<br>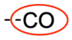<br>BCP-3 — The two π components in the carbonyl fragment are π bonds matching BCP-3<br>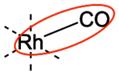<br>BCP-7 — The dative coordination of the carbonyl ligand is a component matching the simple σ bond BCP-1, but the back-donation from the metal into the π* of CO matches BCP-7.	☒ BCP-1<br>☐ BCP-2<br>☒ BCP-3<br>☐ BCP-4<br>☐ BCP-5<br>☐ BCP-6<br>☒ BCP-7<br>☐ BCP-8	N.A.	Octahedral
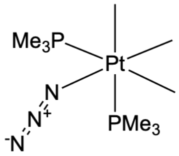<br>ABEKIF	☒ BCP-1<br>☐ BCP-2<br>☐ BCP-3<br>☐ BCP-4<br>☐ BCP-5<br>☐ BCP-6<br>☒ BCP-7<br>☐ BCP-8	N.A.	Octahedral
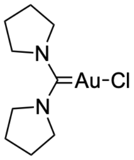<br>APUFEX<br>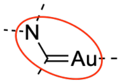<br>BCP-7 — The carbene donates to the metal forming a σ bond component (BCP-1), but the back-donation into the π system of the N-substituted carbene matches BCP-7.	☒ BCP-1<br>☒ BCP-2<br>☐ BCP-3<br>☐ BCP-4<br>☐ BCP-5<br>☐ BCP-6<br>☒ BCP-7<br>☐ BCP-8	N.A.	Linear

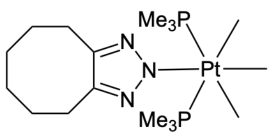 ABEHAU	☒ BCP-1<br>☒ BCP-2<br>☐ BCP-3<br>☐ BCP-4<br>☐ BCP-5<br>☐ BCP-6<br>☐ BCP-7<br>☒ BCP-8	Fused rings (saturated & aromatic)	Octahedral
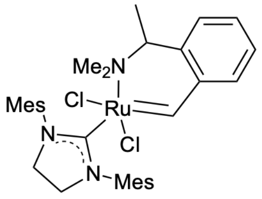 TITTUO	☒ BCP-1<br>☒ BCP-2<br>☐ BCP-3<br>☐ BCP-4<br>☐ BCP-5<br>☐ BCP-6<br>☒ BCP-7<br>☒ BCP-8	Fused rings (aromatic & metal-containing, unsaturated)	Distorted square pyramidal
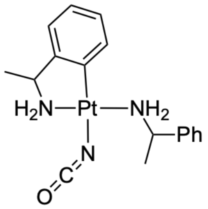 EGEYOG	☒ BCP-1<br>☒ BCP-2<br>☐ BCP-3<br>☐ BCP-4<br>☐ BCP-5<br>☐ BCP-6<br>☒ BCP-7<br>☒ BCP-8	Fused rings (aromatic & metal-containing)	Square planar
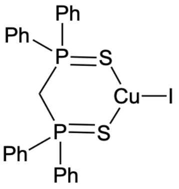 ABOBUP	☒ BCP-1<br>☒ BCP-2<br>☐ BCP-3<br>☐ BCP-4<br>☐ BCP-5<br>☐ BCP-6<br>☐ BCP-7<br>☒ BCP-8	N.A.	Trigonal planar
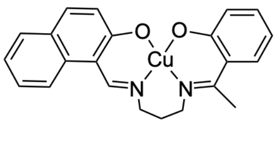 XIDTOW	☒ BCP-1<br>☒ BCP-2<br>☐ BCP-3<br>☐ BCP-4<br>☐ BCP-5<br>☐ BCP-6<br>☐ BCP-7<br>☒ BCP-8	Fused rings (aromatic, unsaturated, saturated, metal-containing)	Square planar

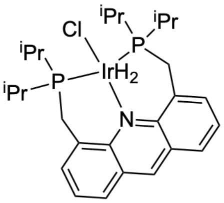<br>NIVHEJ	☒ BCP-1<br>☒ BCP-2<br>☐ BCP-3<br>☐ BCP-4<br>☐ BCP-5<br>☐ BCP-6<br>☐ BCP-7<br>☒ BCP-8	Fused rings (aromatic, metal-containing)	Octahedral
**Category: Multihapto**			
**2D Structure,[a] CSD Refcode, and Commented Examples of BCP Matches**	**BCP Matches**	**Compound Patterns**	**Metal Geometry**
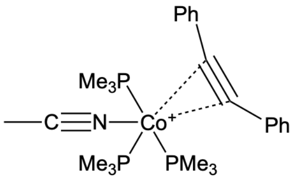<br>ACNCOB10<br>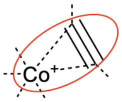<br>BCP-5<br>The η^2(C=C)Co unit matches BCP-5 using one of the π components of the triple bond. The other component is part of the π system involving the phenyl ligands, thus matching BCP-7 with a bent triad. BCP-7 is matched also by the trigonal C−Co−C triad via the back-donation component of η^2(C=C)Co.	☒ BCP-1<br>☒ BCP-2<br>☒ BCP-3<br>☐ BCP-4<br>☒ BCP-5<br>☐ BCP-6<br>☒ BCP-7<br>☒ BCP-8	N.A.	Trigonal bipyramidal
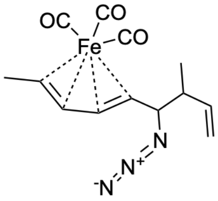<br>TACXUQ<br>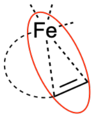<br>BCP-6<br>Each η^2(C=C)Fe unit matches BCP-6 instead of BCP-5 because of the cyclicity resulting from the other unit.	☒ BCP-1<br>☒ BCP-2<br>☐ BCP-3<br>☐ BCP-4<br>☐ BCP-5<br>☒ BCP-6<br>☒ BCP-7<br>☐ BCP-8	N.A.	Tetrahedral

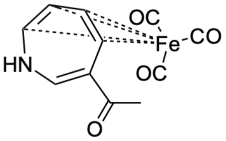<br>ACAZFE	☒ BCP-1<br>☒ BCP-2<br>☒ BCP-3<br>☐ BCP-4<br>☐ BCP-5<br>☒ BCP-6<br>☒ BCP-7<br>☒ BCP-8	N.A.	Tetrahedral
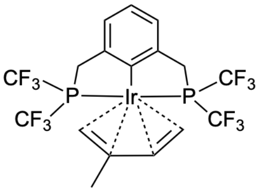<br>ADUPAS	☒ BCP-1<br>☒ BCP-2<br>☐ BCP-3<br>☐ BCP-4<br>☐ BCP-5<br>☒ BCP-6<br>☐ BCP-7<br>☒ BCP-8	Fused rings (aromatic & metal-containing)	Distorted square pyramidal
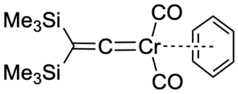<br>DAJLAC<br>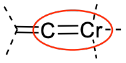<br>BCP-3<br>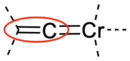<br>BCP-3<br>Both sides of the metal-vinylidene (i.e., a metal-allene) match BCP-3 because they are orthogonal to each other.	☒ BCP-1<br>☐ BCP-2<br>☒ BCP-3<br>☐ BCP-4<br>☐ BCP-5<br>☒ BCP-6<br>☐ BCP-7<br>☒ BCP-8	Cumulene involving metal	Tetrahedral
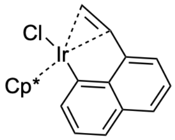<br>OFOWIS	☒ BCP-1<br>☒ BCP-2<br>☐ BCP-3<br>☐ BCP-4<br>☐ BCP-5<br>☒ BCP-6<br>☐ BCP-7<br>☒ BCP-8	Fused rings (aromatic & dative chelating ring)	Tetrahedral
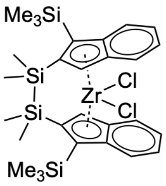<br>GUDQOL	☒ BCP-1<br>☒ BCP-2<br>☐ BCP-3<br>☐ BCP-4<br>☐ BCP-5<br>☒ BCP-6<br>☐ BCP-7<br>☒ BCP-8	Polydentate multihapto ligand	Tetrahedral

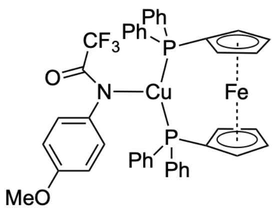<br>AFIXEV	☒ BCP-1<br>☒ BCP-2<br>☐ BCP-3<br>☐ BCP-4<br>☐ BCP-5<br>☒ BCP-6<br>☒ BCP-7<br>☒ BCP-8	Bimetallic complex	Linear, Trigonal planar
**Category: MXY**			
**2D Structure,[a] CSD Refcode, and Commented Examples of BCP Matches**	**BCP Matches**	**Compound Patterns**	**Metal Geometry**
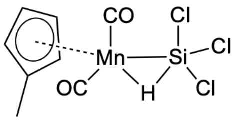<br>CATSUL<br>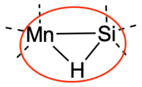<br>BCP-5<br>Three-center two-electron bond representing the α-agostic interaction where the Si−H bond shares electrons with the metal center.	☒ BCP-1<br>☐ BCP-2<br>☒ BCP-3<br>☐ BCP-4<br>☒ BCP-5<br>☒ BCP-6<br>☒ BCP-7<br>☒ BCP-8	N.A.	Distorted tetrahedral
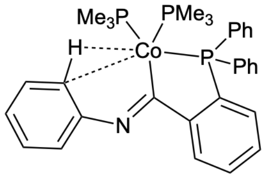<br>HESMUQ01<br>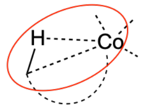<br>BCP-6<br>δ-agostic interaction where the C−H bond donated into the metal forming a σ symmetry component that is cyclic because of imine link.	☒ BCP-1<br>☒ BCP-2<br>☐ BCP-3<br>☐ BCP-4<br>☐ BCP-5<br>☒ BCP-6<br>☐ BCP-7<br>☒ BCP-8	Fused rings (aromatic, metal-containing with agostic bonds)	Distorted trigonal bipyramidal
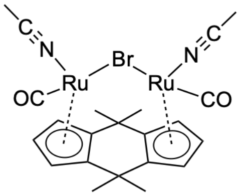<br>AKOQOH<br>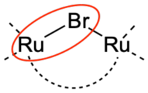<br>BCP-2<br>The bridging Br atom can be seen as a covalent ligand to one Ru and a dative ligand to the other Ru center, thus matching BCP-2 on either side.	☒ BCP-1<br>☒ BCP-2<br>☒ BCP-3<br>☐ BCP-4<br>☐ BCP-5<br>☒ BCP-6<br>☒ BCP-7<br>☒ BCP-8	Multi-bridged bimetallic complex	Tetrahedral

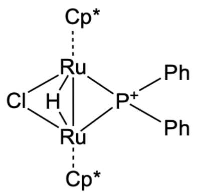<br>ADARUT<br>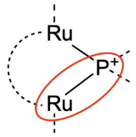<br>BCP-2<br>The bridging phosphido ligand is interpreted as bonded to each Ru with a σ bond, which is cyclic by virtue of the other two bridging ligands, hence matching BCP-2.<br>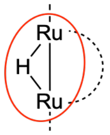<br>BCP-6<br>The bonding of the bridging H is interpreted as a two-electron three-centers bond involving both Ru centers, in a σ-symmetry bond, that is cyclic, thus matching BCP-6.	☒ BCP-1<br>☒ BCP-2<br>☐ BCP-3<br>☐ BCP-4<br>☐ BCP-5<br>☒ BCP-6<br>☐ BCP-7<br>☒ BCP-8	Multi-bridged bimetallic complex with 3c-2e bond	Distorted tetrahedral

Category: La/Ac

2D Structure,[a] CSD Refcode, and Commented Examples of BCP Matches	BCP Matches	Compound Patterns	Metal Geometry
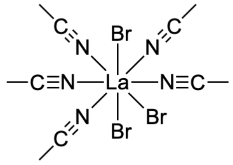<br>ABEVAG	☒ BCP-1<br>☐ BCP-2<br>☒ BCP-3<br>☐ BCP-4<br>☐ BCP-5<br>☐ BCP-6<br>☒ BCP-7<br>☐ BCP-8	N.A.	Distorted octa-coordinated
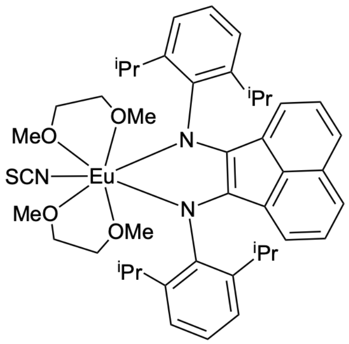<br>AFECIA	☒ BCP-1<br>☒ BCP-2<br>☐ BCP-3<br>☐ BCP-4<br>☐ BCP-5<br>☐ BCP-6<br>☒ BCP-7<br>☒ BCP-8	Fused rings (aromatic, unsaturated, saturated and metal-containing), large coordination number	Distorted hepta-coordinated

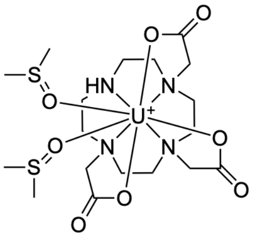<br>ACOVUL	☒ BCP-1<br>☒ BCP-2<br>☒ BCP-3<br>☐ BCP-4<br>☐ BCP-5<br>☐ BCP-6<br>☐ BCP-7<br>☒ BCP-8	Fused rings, macrocyclic polydentate ligand, large coordination number	Distorted nona-coordinated

Category: OS

**2D Structure,[a] CSD Refcode, and Commented Examples of BCP Matches**	**BCP Matches**	**Compound Patterns**	**Metal Geometry**
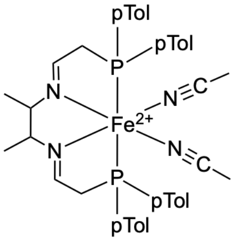<br>ABAYAF	☒ BCP-1<br>☒ BCP-2<br>☒ BCP-3<br>☒ BCP-4<br>☐ BCP-5<br>☐ BCP-6<br>☒ BCP-7<br>☐ BCP-8	Macrocyclic polydentate ligand, charged metal	Distorted octahedral
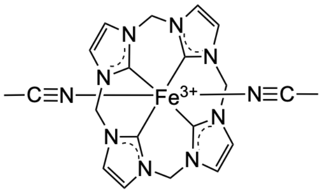<br>RULJAM	☒ BCP-1<br>☒ BCP-2<br>☒ BCP-3<br>☐ BCP-4<br>☐ BCP-5<br>☐ BCP-6<br>☒ BCP-7<br>☒ BCP-8	Macrocyclic polydentate ligand, charged metal	Octahedral

[a]Abbreviations used in 2D representations: Me, Methyl; ^{i}Pr: isopropyl; Et, Ethyl; Ph, Phenyl; Mes: Mesityl; pTol: para-tolyl; Cp*: pentamethylcyclopentadienyl.

S.2. Starting Populations and Coverage Analysis

For each benchmark target, the starting population is defined as the subset of molecules from the overall dataset that exhibit an HSR similarity to the target below a specified threshold. The rationale for imposing this threshold is to ensure that the initial chemical space does not contain any molecule similar to the target, thereby presenting a realistic and non-trivial challenge for generative algorithms.

To investigate the impact of the threshold choice, we evaluated three different similarity thresholds 0.3, 0.4 and 0.5. The size of the resulting starting population for each target at these thresholds is shown in Figure S6.

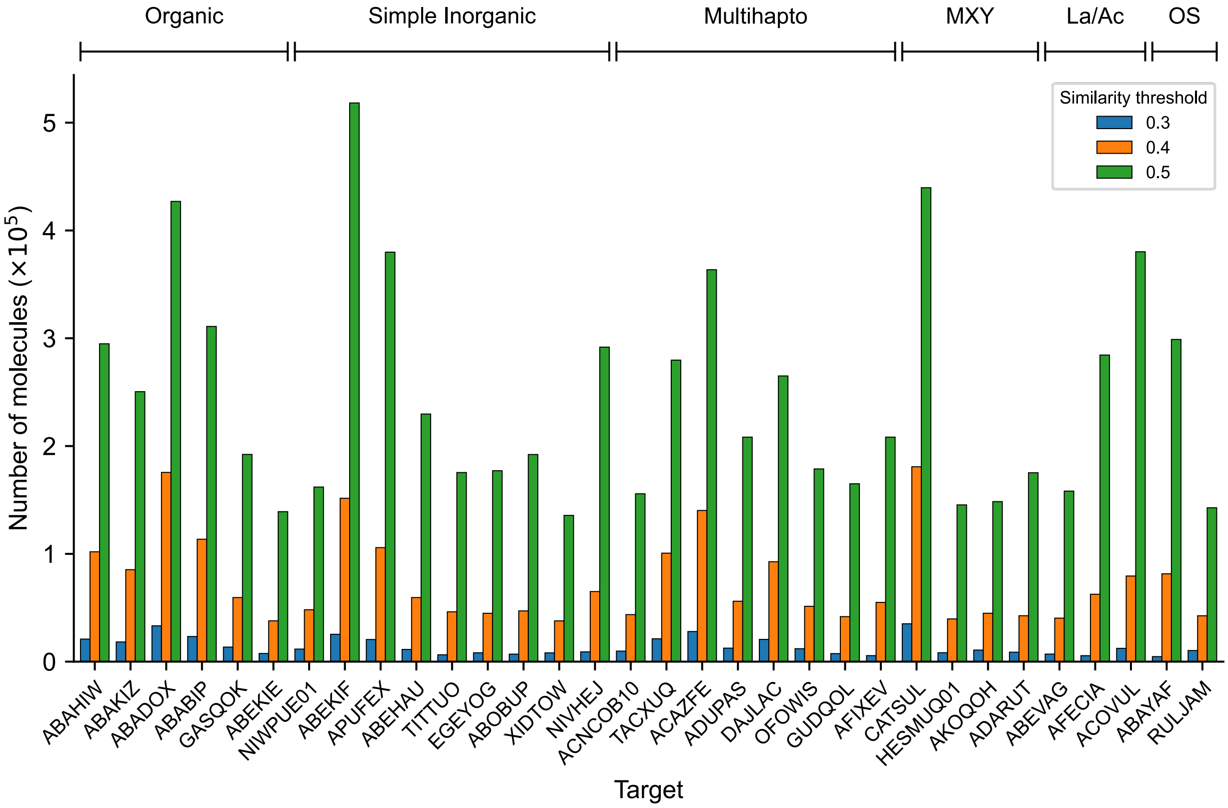

Figure S6. Number of molecules in the starting populations of each target at three different similarity thresholds: 0.3, 0.4, and 0.5. Targets are grouped by category (labels above the plot) as explained in Section S.1.3.

In addition to their sizes, we characterized the composition of the starting populations by inspecting the distribution of HSR similarities to the target. Figure S7 reports these distributions for the three thresholds (0.3, 0.4, 0.5). For each threshold, the histograms are obtained by pooling together the similarity values from all benchmark targets into a single aggregated dataset, so that the plots reflect the overall similarity profile of the entire starting population rather than individual targets. By construction, all histograms are right-truncated at the chosen threshold, showing a pronounced right-skew, with density piling up just below the cutoff. For more detailed per-target distributions, see 3DOpt repository.

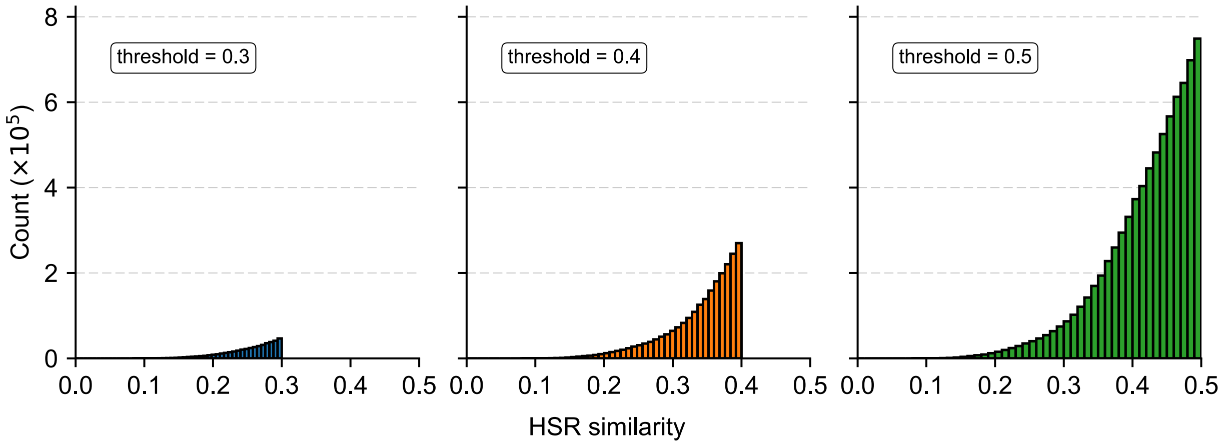

Figure S7. Aggregated HSR similarity distributions for the starting populations at three thresholds (0.3, 0.4, 0.5), obtained by pooling similarities from all benchmark targets.

To assess whether a given starting population contains the necessary building blocks to reconstruct the target molecule, we analyze the presence of *Connected Atom Environments* (CAEs), defined as the substructure formed by a central atom and all its directly bonded neighbors, specified by their 3D coordinates and relevant atomic features (e.g., proton number, formal charge).

For each target, we enumerate all CAEs and, for each starting population, determine whether an equivalent fragment (i.e., CAE) can be found. The classifications are as follows:

i. **Biatomic CAEs**

- **Match:** A fragment in the starting population has the same element pair as the target CAE and a bond length within 0.1 Å of the target value.
- **Distorted match:** The element pair exists, but the bond length differs by more than 0.1 Å.
- **Non-match:** No fragment with the same element pair is present in the population.

ii. **Polyatomic CAEs**

- **Match:** A fragment with the same formula achieves an HSR similarity ≥ 0.98 to the target CAE.
- **Distorted match:** The formula is present, but HSR similarity < 0.98.
- **Non-match:** No fragment with the same formula is present.

Using the above classification, we define *coverage* for each target and threshold as the percentage of the target's CAEs that have at least one match. We also report the *total coverage* (sum of matches and distorted matches), representing the fraction of CAEs present in either perfect or geometrically distorted form.

Figure S8, Figure S9, and Figure S10 summarize the percentages of matches, distorted matches, and non-matches for each target at similarity thresholds 0.3, 0.4, and 0.5 respectively. Table S2 reports the raw classification numbers for all three cases.

These results quantify the fragment diversity in each starting population and help inform the choice of threshold for the benchmark.

Considering these results, a threshold of 0.5 was chosen to extract the starting populations for the 3DOpt benchmark tasks.

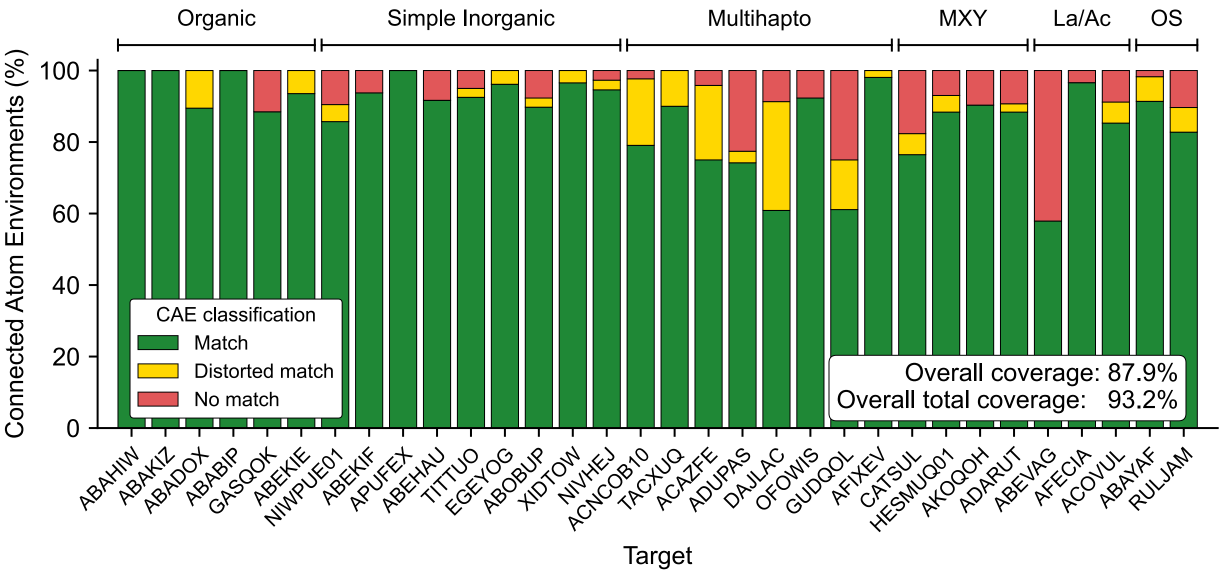

Figure S8. Per-target proportions of CAE matches, distorted matches, and non-matches at similarity threshold 0.3, with overall coverage statistics.

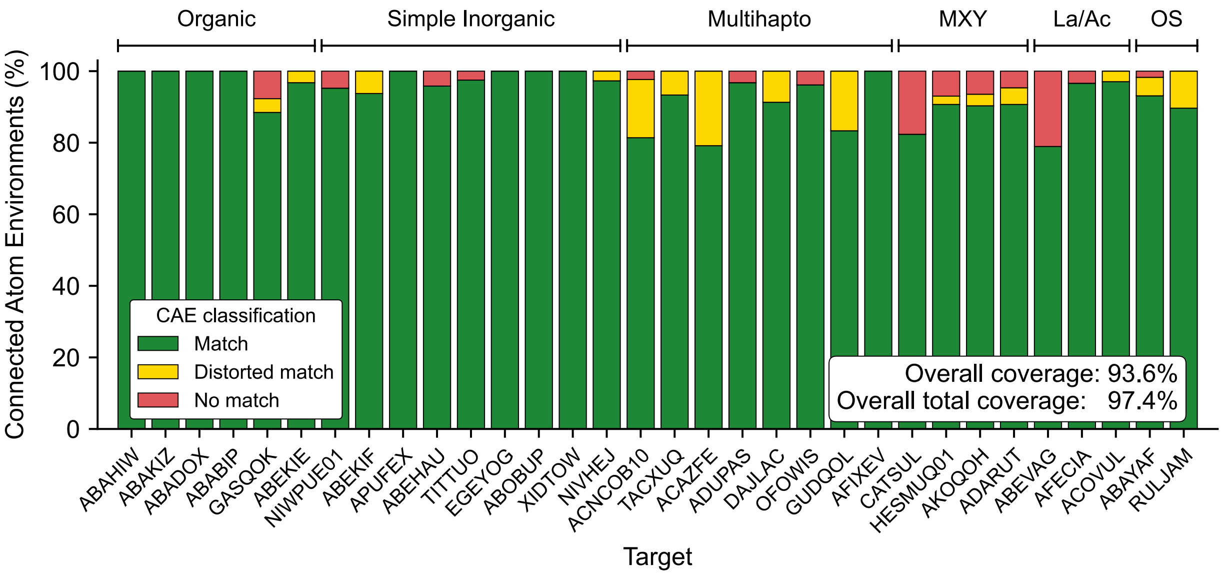

Figure S9. Per-target proportions of CAE matches, distorted matches, and non-matches at similarity threshold 0.4, with overall coverage statistics.

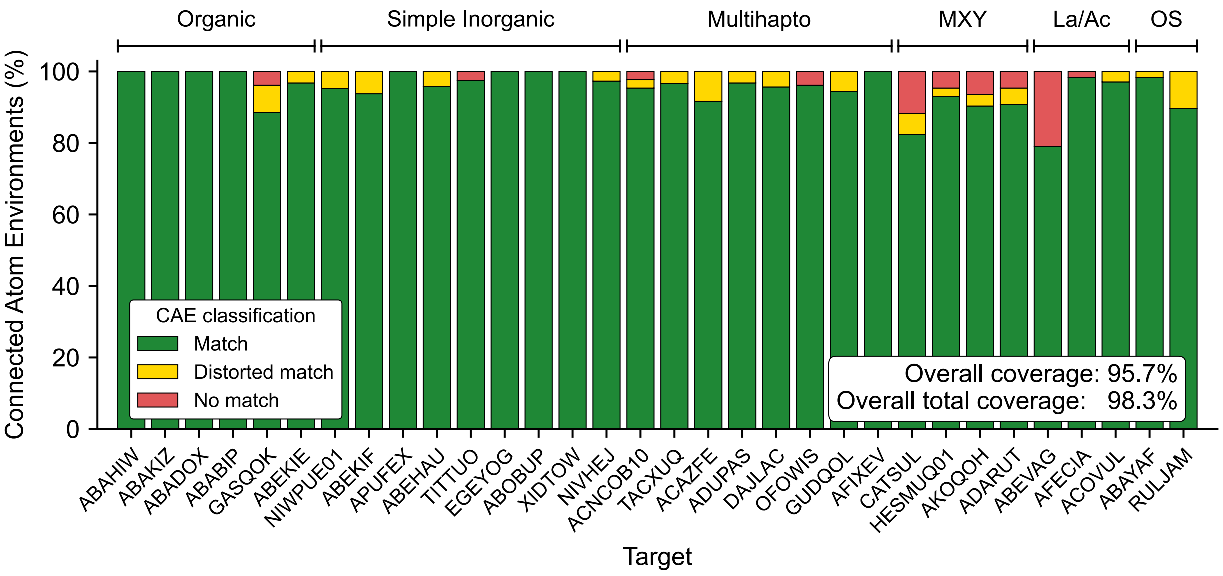

Figure S10. Per-target proportions of CAE matches, distorted matches, and non-matches at similarity threshold 0.5, with overall coverage statistics.

Table S2. Number of matches, distorted matches and non matches CAEs for each similarity threshold, 0.3, 0.4, 0,5. M = matched, D = distorted match, N = No match.

Target	Total target CAE	Similarity threshold								
		0.3			0.4			0.5		
		M	D	N	M	D	N	M	D	N
ABAHIW	16	16	0	0	16	0	0	16	0	0
ABAKIZ	17	17	0	0	17	0	0	17	0	0
ABADOX	19	17	2	0	19	0	0	19	0	0
ABABIP	22	22	0	0	22	0	0	22	0	0
GASQOK	26	23	0	3	23	1	2	23	2	1
ABEKIE	31	29	2	0	30	1	0	30	1	0
NIWPUE01	21	18	1	2	20	0	1	20	1	0
ABEKIF	16	15	0	1	15	1	0	15	1	0
APUFEX	13	13	0	0	13	0	0	13	0	0
ABEHAU	24	22	0	2	23	0	1	23	1	0
TITTUO	40	37	1	2	39	0	1	39	0	1
EGEYOG	26	25	1	0	26	0	0	26	0	0
ABOBUP	39	35	1	3	39	0	0	39	0	0
XIDTOW	29	28	1	0	29	0	0	29	0	0
ACNCOB10	43	34	8	1	35	7	1	41	1	1
TACXUQ	30	27	3	0	28	2	0	29	1	0
ACAZFE	24	18	5	1	19	5	0	22	2	0

NIVHEJ	37	35	1	1	36	1	0	36	1	0
ADUPAS	31	23	1	7	30	0	1	30	1	0
DAJLAC	23	14	7	2	21	2	0	22	1	0
OFOWIS	26	24	0	2	25	0	1	25	0	1
CATSUL	17	13	1	3	14	0	3	14	1	2
HESMUQ01	43	38	2	3	39	1	3	40	1	2
GUDQOL	36	22	5	9	30	6	0	34	2	0
ABEVAG	19	11	0	8	15	0	4	15	0	4
AKOQOH	31	28	0	3	28	1	2	28	1	2
ADARUT	43	38	1	4	39	2	2	39	2	2
AFECIA	59	57	0	2	57	0	2	58	0	1
ACOVUL	34	29	2	3	33	1	0	33	1	0
AFIXEV	52	51	1	0	52	0	0	52	0	0
ABAYAF	58	53	4	1	54	3	1	57	1	0
RULJAM	29	24	2	3	26	3	0	26	3	0

S.3. Computational Details

All software, data, and results required to reproduce the analyses, figures, and benchmark experiments in this work are publicly available through the following resources:

- **MolScore** (https://github.com/MorganCThomas/MolScore):

  Provides the implementation of the 3DOpt benchmark as part of the MolScore molecular design framework.

- **MolScore Baselines:** (https://github.com/MorganCThomas/MolScore_baselines):

  Supplies scripts and tools to run baseline generative methods (RandomSampler and ChemGE) on the 3DOpt benchmark, as well as code for reproducing the main results and figures.

- **3DOpt** (https://github.com/marcellocostamagna/3DOpt):

  Contains supporting scripts, workflows, and selected analyses for data preparation, target selection, coverage analysis, and additional supplementary figures.

- **Baseline Results Archive** (https://zenodo.org/records/16602642):

  The complete archive of all baseline results (including all runs for every method and generator) reported in this work is available on Zenodo. Each compressed archive contains the full set of output files and logs for reproducibility and further analysis.

S.3.1. Baseline Methods

The baseline algorithms evaluated in this study—Random Sampler and ChemGE—were implemented and executed using the MolScore Baselines repository. All results discussed in this work can be accessed in the Zenodo results archive. Workflows, scripts, and utilities for analyzing and visualizing these results (including the generation of Figures 4, 7 and 8 in the main paper) are available in the folder *3DOpt_baseline_results* in the 3DOpt repository.

S.3.1.1. SMILES-to-3D pipeline settings

The SMILES-to-3D pipelines used in the baseline methods and named CCDC, Open Babel, and RDKit are available in the MolScore repository (https://github.com/MorganCThomas/MolScore) within the HSR similarity scoring function (molscore/scoring_functions/hsr_sim.py). Here is an overview of each pipeline settings:

- CCDC pipeline:[9] i) generation of the molecular object from the given SMILES, ii) generation of one conformer, which embeds steps of addition of missing Hydrogen atoms generation of 3D coordinates, energy minimization with the Tripos force field, and sampling of conformational space. 10 seconds timeout imposed.
- OpenBabel pipeline:[10] i) generation of the molecular object from the given SMILES, ii) addition of explicit Hydrogen atoms, iii) generation of 3D coordinate with crude energy minimization using the default MMFF94 forcefield, and iv) refined energy minimization (i.e., local geometry optimization) again using the default MMFF94 forcefield. No timeout imposed.
- RDKit pipeline:[11] i) generation of the molecular object from the given SMILES using, ii) addition of explicit Hydrogen atoms, iii) generation of 3D coordinate, and iv) energy minimization (i.e., local geometry optimization) using the default MMFF94 forcefield. No timeout imposed.

S.3.2. Data Set Generation

The initial set of molecular structures, from which all 3DOpt targets and starting populations were derived, was obtained from the Cambridge Structural Database (CSD).[12,13] Structure retrieval, filtering, and preparation were performed using the scripts in the *Filtering* folder of the 3DOpt repository.

S.3.3. Targets Selection

Benchmark targets were selected according to the strategy described in Section S.1. Statistics for the metal-containing targets (Section S.1.3) can be reproduced using the scripts in the *Metal_targets_statistics* folder of the 3DOpt repository. 2D diagrams for all targets, as reported in Table S1, can be generated from the *3DOpt_configuration* folder in the same repository.

S.3.4. Starting Populations and Coverage Analysis

The starting populations and their initial analysis (Figure **S6**) can be reproduced using the scripts and workflows provided in the *Starting_populations* folder of the 3DOpt repository.

The generation and analysis of Connected Atom Environments (CAEs), as reported in Section S.2, are documented in the *CAEs* and *CAEs_analysis* folders of the same repository.

S.3.5. Generators analysis

The results presented in Figures 5 and 6 of the main paper can be found in the *Generator_analysis* folder of the 3DOpt repository along with the scripts and workflow to reproduce them.

S.3.5.1. Explicit-hydrogen SMILES analysis

To assess the impact of using explicit-hydrogen SMILES, we attempted to generate the structures of the 32 benchmark targets with all three conformer generation methods (CCDC, RDKit, and Open Babel), using both implicit- and explicit-hydrogen SMILES. For each combination, 100 runs with different random seeds were performed. Success rates with explicit-hydrogen SMILES were substantially lower across all methods, as summarized in Table S3. As discussed in the main text, this observation motivated our choice to retain implicit-hydrogen SMILES throughout the study. The scripts and workflows to reproduce these results are provided in the *Generator_analysis* folder of the 3DOpt repository.

Table S3. Comparison of conformer generation success rates for implicit vs. explicit SMILES across methods.

SMILES	Succes Rate (%)		
	CCDC	RDKit	OBabel
Implicit H	88	53	78
Explicit H	78	44	66

S.4. Computational Data

S.4.1. Baselines Results per Run

S.4.1.1. Random Sampler from CCDC entries

**Task**	**Run**									
	**1**	**2**	**3**	**4**	**5**	**6**	**7**	**8**	**9**	**10**
ABAHIW	0.50	0.50	0.50	0.50	0.50	0.50	0.50	0.49	0.50	0.50
ABAKIZ	0.50	0.50	0.50	0.50	0.50	0.50	0.50	0.50	0.50	0.50
ABADOX	0.49	0.49	0.49	0.49	0.49	0.49	0.49	0.50	0.50	0.50

ABABIP	0.50	0.50	0.50	0.50	0.50	0.50	0.50	0.50	0.50	0.50
GASQOK	0.50	0.50	0.50	0.49	0.50	0.50	0.50	0.49	0.50	0.50
ABEKIE	0.50	0.50	0.50	0.50	0.50	0.50	0.49	0.50	0.50	0.50
NIWPUE01	0.50	0.50	0.50	0.50	0.50	0.50	0.50	0.50	0.50	0.50
ABEKIF	0.49	0.50	0.50	0.50	0.50	0.49	0.49	0.50	0.49	0.50
APUFEX	0.50	0.50	0.50	0.50	0.50	0.49	0.50	0.50	0.49	0.50
ABEHAU	0.50	0.50	0.50	0.50	0.49	0.49	0.50	0.50	0.50	0.50
TITTUO	0.50	0.50	0.50	0.50	0.50	0.50	0.50	0.50	0.50	0.50
EGEYOG	0.50	0.50	0.50	0.50	0.50	0.50	0.50	0.50	0.50	0.50
ABOBUP	0.50	0.50	0.50	0.50	0.50	0.50	0.49	0.50	0.50	0.50
XIDTOW	0.50	0.50	0.50	0.50	0.50	0.50	0.50	0.50	0.50	0.50
ACNCOB10	0.50	0.49	0.50	0.50	0.50	0.50	0.50	0.49	0.50	0.50
TACXUQ	0.50	0.49	0.49	0.49	0.50	0.50	0.50	0.50	0.49	0.50
ACAZFE	0.49	0.49	0.50	0.50	0.50	0.49	0.49	0.50	0.49	0.50
NIVHEJ	0.50	0.50	0.50	0.50	0.50	0.50	0.50	0.50	0.50	0.50
ADUPAS	0.50	0.50	0.50	0.50	0.50	0.50	0.50	0.49	0.50	0.50
DAJLAC	0.49	0.50	0.50	0.50	0.50	0.50	0.50	0.50	0.50	0.49
OFOWIS	0.49	0.50	0.50	0.50	0.50	0.50	0.50	0.50	0.50	0.49
CATSUL	0.49	0.49	0.50	0.50	0.49	0.49	0.50	0.49	0.50	0.49
HESMUQ01	0.50	0.49	0.50	0.50	0.50	0.50	0.49	0.50	0.50	0.50
GUDQOL	0.50	0.50	0.50	0.50	0.50	0.50	0.50	0.50	0.50	0.50
ABEVAG	0.50	0.50	0.50	0.50	0.50	0.50	0.50	0.50	0.50	0.50
AKOQOH	0.50	0.50	0.50	0.50	0.50	0.50	0.50	0.50	0.50	0.49
ADARUT	0.50	0.50	0.50	0.50	0.50	0.50	0.50	0.50	0.50	0.50
AFECIA	0.50	0.50	0.50	0.50	0.50	0.50	0.50	0.50	0.50	0.50
ACOVUL	0.50	0.50	0.50	0.50	0.50	0.50	0.50	0.50	0.50	0.50
AFIXEV	0.49	0.50	0.50	0.50	0.50	0.50	0.50	0.50	0.49	0.49
ABAYAF	0.50	0.50	0.50	0.50	0.50	0.49	0.50	0.50	0.50	0.50
RULJAM	0.49	0.50	0.50	0.49	0.50	0.50	0.49	0.50	0.50	0.50

S.4.1.2. Random Sampler with CCDC

Task	Run									
	1	2	3	4	5	6	7	8	9	10
ABAHIW	0.55	0.58	0.55	0.54	0.54	0.53	0.61	0.54	0.55	0.53
ABAKIZ	0.53	0.57	0.54	0.56	0.53	0.52	0.55	0.55	0.57	0.61
ABADOX	0.54	0.55	0.53	0.56	0.53	0.53	0.53	0.52	0.53	0.51
ABABIP	0.52	0.55	0.54	0.56	0.54	0.53	0.52	0.55	0.51	0.55

GASQOK	0.55	0.58	0.54	0.60	0.52	0.55	0.59	0.59	0.57	0.66
ABEKIE	0.55	0.53	0.56	0.58	0.59	0.53	0.64	0.60	0.55	0.66
NIWPUE01	0.56	0.63	0.55	0.56	0.60	0.62	0.61	0.56	0.60	0.61
ABEKIF	0.51	0.55	0.55	0.52	0.52	0.52	0.53	0.53	0.54	0.50
APUFEX	0.56	0.56	0.58	0.57	0.56	0.53	0.60	0.53	0.52	0.54
ABEHAU	0.55	0.55	0.57	0.53	0.57	0.54	0.54	0.63	0.57	0.56
TITTUO	0.53	0.56	0.59	0.53	0.55	0.53	0.60	0.57	0.57	0.53
EGEYOG	0.55	0.55	0.57	0.55	0.61	0.54	0.59	0.60	0.60	0.59
ABOBUP	0.57	0.56	0.60	0.53	0.58	0.61	0.63	0.60	0.58	0.56
XIDTOW	0.60	0.54	0.59	0.61	0.54	0.60	0.55	0.55	0.61	0.56
ACNCOB10	0.59	0.54	0.54	0.61	0.58	0.55	0.52	0.60	0.56	0.53
TACXUQ	0.58	0.56	0.51	0.54	0.61	0.55	0.58	0.57	0.52	0.52
ACAZFE	0.57	0.52	0.52	0.51	0.58	0.56	0.53	0.52	0.52	0.51
NIVHEJ	0.55	0.53	0.57	0.55	0.54	0.58	0.56	0.57	0.56	0.53
ADUPAS	0.57	0.54	0.55	0.53	0.61	0.57	0.54	0.55	0.55	0.55
DAJLAC	0.54	0.54	0.53	0.55	0.54	0.56	0.55	0.63	0.63	0.52
OFOWIS	0.55	0.57	0.55	0.55	0.54	0.53	0.55	0.61	0.56	0.55
CATSUL	0.54	0.53	0.53	0.56	0.52	0.50	0.54	0.51	0.54	0.52
HESMUQ01	0.55	0.60	0.61	0.55	0.54	0.56	0.55	0.53	0.58	0.55
GUDQOL	0.53	0.57	0.53	0.53	0.60	0.57	0.54	0.60	0.54	0.54
ABEVAG	0.56	0.60	0.57	0.59	0.54	0.53	0.53	0.61	0.58	0.63
AKOQOH	0.58	0.57	0.53	0.57	0.63	0.55	0.59	0.55	0.56	0.56
ADARUT	0.58	0.53	0.56	0.54	0.61	0.55	0.55	0.59	0.54	0.55
AFECIA	0.56	0.53	0.54	0.55	0.54	0.53	0.52	0.56	0.58	0.56
ACOVUL	0.52	0.54	0.56	0.62	0.58	0.53	0.53	0.55	0.54	0.55
AFIXEV	0.57	0.54	0.53	0.55	0.59	0.53	0.62	0.62	0.54	0.61
ABAYAF	0.57	0.54	0.62	0.61	0.57	0.55	0.58	0.55	0.54	0.56
RULJAM	0.55	0.60	0.60	0.63	0.61	0.55	0.55	0.54	0.57	0.64

S.4.1.3. Random Sampler with RDKit

Task	Run									
	1	2	3	4	5	6	7	8	9	10
ABAHIW	0.58	0.54	0.59	0.50	0.54	0.52	0.57	0.54	0.53	0.53
ABAKIZ	0.51	0.52	0.56	0.53	0.54	0.52	0.56	0.55	0.52	0.51
ABADOX	0.52	0.50	0.59	0.51	0.54	0.52	0.50	0.51	0.56	0.55
ABABIP	0.50	0.55	0.54	0.56	0.54	0.54	0.54	0.50	0.53	0.58
GASQOK	0.57	0.60	0.58	0.60	0.59	0.59	0.56	0.58	0.55	0.57

ABEKIE	0.57	0.52	0.54	0.55	0.57	0.61	0.59	0.59	0.62	0.58
NIWPUE01	0.56	0.58	0.59	0.56	0.57	0.61	0.56	0.56	0.60	0.58
ABEKIF	0.52	0.52	0.53	0.52	0.51	0.51	0.56	0.51	0.51	0.51
APUFEX	0.56	0.53	0.54	0.52	0.53	0.51	0.55	0.53	0.51	0.53
ABEHAU	0.52	0.57	0.52	0.52	0.58	0.52	0.58	0.64	0.57	0.57
TITTUO	0.56	0.56	0.56	0.53	0.58	0.59	0.53	0.60	0.61	0.61
EGEYOG	0.59	0.55	0.56	0.55	0.57	0.52	0.55	0.54	0.60	0.57
ABOBUP	0.54	0.54	0.51	0.59	0.55	0.53	0.55	0.59	0.54	0.57
XIDTOW	0.54	0.57	0.55	0.55	0.54	0.64	0.55	0.55	0.61	0.56
ACNCOB10	0.52	0.53	0.54	0.55	0.58	0.57	0.54	0.57	0.58	0.60
TACXUQ	0.55	0.58	0.56	0.54	0.55	0.53	0.52	0.52	0.51	0.53
ACAZFE	0.50	0.51	0.54	0.52	0.53	0.50	0.54	0.53	0.52	0.50
NIVHEJ	0.57	0.54	0.54	0.56	0.55	0.54	0.53	0.57	0.54	0.54
ADUPAS	0.55	0.59	0.54	0.56	0.53	0.55	0.54	0.62	0.55	0.64
DAJLAC	0.54	0.54	0.55	0.53	0.59	0.54	0.57	0.58	0.54	0.54
OFOWIS	0.56	0.60	0.58	0.54	0.55	0.56	0.54	0.56	0.57	0.58
CATSUL	0.54	0.60	0.51	0.56	0.57	0.50	0.54	0.56	0.51	0.53
HESMUQ01	0.54	0.53	0.53	0.57	0.58	0.57	0.60	0.53	0.55	0.55
GUDQOL	0.56	0.57	0.53	0.53	0.55	0.54	0.57	0.54	0.55	0.54
ABEVAG	0.56	0.58	0.56	0.56	0.57	0.56	0.56	0.56	0.56	0.60
AKOQOH	0.54	0.64	0.56	0.54	0.58	0.57	0.54	0.51	0.57	0.58
ADARUT	0.55	0.52	0.53	0.53	0.52	0.58	0.53	0.58	0.54	0.57
AFECIA	0.55	0.54	0.53	0.54	0.53	0.53	0.54	0.52	0.58	0.53
ACOVUL	0.53	0.50	0.55	0.59	0.52	0.52	0.54	0.52	0.55	0.53
AFIXEV	0.58	0.56	0.60	0.55	0.52	0.57	0.53	0.51	0.53	0.59
ABAYAF	0.59	0.54	0.58	0.55	0.55	0.53	0.57	0.54	0.51	0.62
RULJAM	0.55	0.52	0.53	0.55	0.55	0.56	0.53	0.55	0.58	0.54

S.4.1.4. Random Sampler with OBabel

Task	Run									
	1	2	3	4	5	6	7	8	9	10
ABAHIW	0.59	0.67	0.61	0.65	0.68	0.65	0.66	0.70	0.68	0.68
ABAKIZ	0.64	0.69	0.64	0.67	0.64	0.67	0.66	0.71	0.65	0.68
ABADOX	0.64	0.62	0.60	0.64	0.60	0.65	0.62	0.63	0.63	0.71
ABABIP	0.61	0.69	0.67	0.62	0.68	0.64	0.70	0.66	0.67	0.69
GASQOK	0.70	0.73	0.72	0.69	0.64	0.71	0.73	0.73	0.70	0.69
ABEKIE	0.72	0.69	0.68	0.71	0.78	0.72	0.72	0.71	0.70	0.76

NIWPUE01	0.66	0.62	0.65	0.66	0.64	0.68	0.63	0.71	0.69	0.65
ABEKIF	0.58	0.58	0.56	0.56	0.58	0.56	0.61	0.57	0.56	0.57
APUFEX	0.64	0.63	0.56	0.62	0.61	0.60	0.59	0.62	0.59	0.63
ABEHAU	0.62	0.63	0.60	0.63	0.64	0.62	0.69	0.65	0.64	0.63
TITTUO	0.68	0.66	0.71	0.62	0.62	0.67	0.66	0.66	0.65	0.64
EGEYOG	0.66	0.64	0.65	0.66	0.66	0.65	0.71	0.66	0.67	0.61
ABOBUP	0.67	0.67	0.61	0.65	0.65	0.63	0.67	0.67	0.64	0.71
XIDTOW	0.70	0.65	0.68	0.71	0.73	0.73	0.72	0.69	0.74	0.70
ACNCOB10	0.67	0.71	0.71	0.68	0.67	0.71	0.68	0.70	0.68	0.69
TACXUQ	0.70	0.67	0.70	0.74	0.67	0.66	0.67	0.66	0.73	0.69
ACAZFE	0.64	0.64	0.66	0.62	0.60	0.61	0.67	0.62	0.61	0.65
NIVHEJ	0.62	0.60	0.59	0.60	0.60	0.58	0.61	0.61	0.61	0.59
ADUPAS	0.63	0.63	0.65	0.63	0.69	0.62	0.64	0.63	0.62	0.65
DAJLAC	0.63	0.67	0.70	0.71	0.71	0.66	0.64	0.70	0.73	0.65
OFOWIS	0.65	0.65	0.68	0.69	0.69	0.69	0.69	0.67	0.68	0.67
CATSUL	0.56	0.70	0.62	0.64	0.68	0.60	0.66	0.61	0.67	0.61
HESMUQ01	0.75	0.75	0.67	0.72	0.75	0.72	0.65	0.67	0.70	0.67
GUDQOL	0.67	0.69	0.73	0.65	0.67	0.62	0.69	0.56	0.62	0.65
ABEVAG	0.66	0.64	0.69	0.65	0.69	0.66	0.66	0.70	0.67	0.62
AKOQOH	0.71	0.73	0.71	0.73	0.75	0.66	0.73	0.72	0.67	0.74
ADARUT	0.67	0.67	0.65	0.66	0.65	0.65	0.62	0.64	0.64	0.69
AFECIA	0.65	0.61	0.55	0.63	0.60	0.62	0.55	0.63	0.63	0.59
ACOVUL	0.60	0.59	0.59	0.61	0.60	0.57	0.58	0.59	0.59	0.57
AFIXEV	0.68	0.69	0.67	0.68	0.71	0.68	0.67	0.62	0.65	0.63
ABAYAF	0.58	0.57	0.63	0.64	0.61	0.63	0.59	0.67	0.62	0.57
RULJAM	0.68	0.68	0.69	0.68	0.67	0.70	0.66	0.68	0.66	0.72

S.4.1.5. ChemGE with CCDC

Task	Run									
	1	2	3	4	5	6	7	8	9	10
ABAHIW	0.59	0.59	0.59	0.59	0.59	0.59	0.59	0.59	0.59	0.59
ABAKIZ	0.60	0.60	0.61	0.60	0.61	0.61	0.60	0.60	0.60	0.60
ABADOX	0.56	0.56	0.56	0.56	0.56	0.56	0.56	0.56	0.56	0.56
ABABIP	0.59	0.59	0.59	0.59	0.59	0.59	0.59	0.59	0.59	0.59
GASQOK	0.62	0.62	0.62	0.62	0.62	0.62	0.62	0.62	0.62	0.62
ABEKIE	0.57	0.57	0.57	0.57	0.59	0.57	0.57	0.57	0.57	0.58
NIWPUE01	0.59	0.59	0.59	0.59	0.59	0.59	0.59	0.59	0.59	0.59

ABEKIF	0.53	0.53	0.53	0.53	0.53	0.53	0.53	0.53	0.53	0.53
APUFEX	0.54	0.55	0.55	0.55	0.54	0.54	0.56	0.54	0.55	0.55
ABEHAU	0.62	0.62	0.62	0.62	0.62	0.62	0.62	0.62	0.62	0.62
TITTUO	0.57	0.57	0.57	0.57	0.57	0.57	0.57	0.57	0.57	0.57
EGEYOG	0.60	0.60	0.60	0.60	0.60	0.60	0.60	0.60	0.60	0.60
ABOBUP	0.56	0.56	0.56	0.56	0.56	0.56	0.56	0.56	0.56	0.56
XIDTOW	0.64	0.64	0.64	0.64	0.64	0.64	0.64	0.64	0.64	0.64
ACNCOB10	0.58	0.59	0.58	0.59	0.58	0.58	0.58	0.59	0.58	0.58
TACXUQ	0.57	0.57	0.57	0.58	0.57	0.57	0.60	0.57	0.57	0.57
ACAZFE	0.58	0.58	0.58	0.58	0.58	0.58	0.58	0.58	0.59	0.58
NIVHEJ	0.56	0.56	0.56	0.56	0.56	0.56	0.56	0.56	0.56	0.56
ADUPAS	0.60	0.60	0.61	0.60	0.60	0.60	0.60	0.60	0.60	0.60
DAJLAC	0.57	0.57	0.58	0.57	0.57	0.57	0.57	0.57	0.57	0.57
OFOWIS	0.57	0.58	0.57	0.57	0.57	0.58	0.57	0.57	0.57	0.57
CATSUL	0.57	0.57	0.59	0.57	0.57	0.57	0.57	0.57	0.57	0.57
HESMUQ01	0.60	0.61	0.60	0.60	0.60	0.60	0.61	0.60	0.60	0.60
GUDQOL	0.56	0.56	0.55	0.56	0.55	0.57	0.55	0.55	0.56	0.56
ABEVAG	0.62	0.62	0.62	0.62	0.62	0.62	0.62	0.62	0.62	0.62
AKOQOH	0.62	0.62	0.62	0.62	0.62	0.62	0.62	0.62	0.62	0.62
ADARUT	0.57	0.57	0.58	0.58	0.57	0.57	0.57	0.57	0.57	0.58
AFECIA	0.62	0.58	0.58	0.58	0.59	0.58	0.58	0.58	0.58	0.59
ACOVUL	0.55	0.55	0.55	0.55	0.55	0.55	0.55	0.55	0.55	0.55
AFIXEV	0.59	0.59	0.59	0.59	0.60	0.59	0.59	0.59	0.59	0.59
ABAYAF	0.58	0.58	0.58	0.58	0.58	0.58	0.58	0.58	0.58	0.58
RULJAM	0.61	0.61	0.61	0.61	0.61	0.61	0.61	0.61	0.61	0.61

S.4.1.6. ChemGE with RDKit

Task	Run									
	1	2	3	4	5	6	7	8	9	10
ABAHIW	0.56	0.56	0.56	0.56	0.56	0.56	0.59	0.55	0.56	0.54
ABAKIZ	0.57	0.58	0.57	0.57	0.58	0.57	0.57	0.57	0.57	0.57
ABADOX	0.58	0.57	0.58	0.56	0.57	0.58	0.61	0.57	0.56	0.57
ABABIP	0.54	0.58	0.54	0.53	0.55	0.55	0.55	0.53	0.58	0.56
GASQOK	0.60	0.57	0.59	0.58	0.59	0.60	0.59	0.57	0.58	0.61
ABEKIE	0.66	0.58	0.59	0.59	0.60	0.59	0.59	0.59	0.59	0.59
NIWPUE01	0.63	0.57	0.64	0.59	0.58	0.58	0.66	0.63	0.64	0.59
ABEKIF	0.54	0.53	0.52	0.53	0.53	0.53	0.53	0.53	0.53	0.53

APUFEX	0.58	0.58	0.58	0.58	0.58	0.58	0.58	0.59	0.59	0.59
ABEHAU	0.59	0.63	0.61	0.59	0.61	0.59	0.62	0.62	0.56	0.60
TITTUO	0.60	0.62	0.59	0.62	0.60	0.61	0.60	0.57	0.61	0.62
EGEYOG	0.61	0.62	0.62	0.61	0.64	0.63	0.63	0.64	0.60	0.60
ABOBUP	0.57	0.58	0.59	0.57	0.57	0.60	0.57	0.60	0.58	0.57
XIDTOW	0.57	0.56	0.58	0.59	0.60	0.57	0.63	0.58	0.62	0.61
ACNCOB10	0.58	0.58	0.60	0.60	0.61	0.59	0.58	0.59	0.60	0.58
TACXUQ	0.59	0.65	0.62	0.60	0.60	0.61	0.61	0.61	0.60	0.65
ACAZFE	0.63	0.63	0.62	0.56	0.60	0.60	0.60	0.59	0.59	0.59
NIVHEJ	0.58	0.58	0.59	0.56	0.58	0.58	0.57	0.56	0.57	0.58
ADUPAS	0.61	0.61	0.60	0.61	0.61	0.61	0.60	0.61	0.60	0.61
DAJLAC	0.64	0.60	0.60	0.65	0.57	0.57	0.58	0.57	0.56	0.57
OFOWIS	0.60	0.57	0.60	0.59	0.58	0.59	0.60	0.59	0.60	0.63
CATSUL	0.56	0.57	0.59	0.56	0.58	0.58	0.56	0.59	0.56	0.57
HESMUQ01	0.61	0.59	0.62	0.62	0.59	0.66	0.65	0.59	0.60	0.64
GUDQOL	0.60	0.58	0.55	0.59	0.56	0.56	0.61	0.61	0.62	0.62
ABEVAG	0.61	0.63	0.63	0.62	0.64	0.61	0.61	0.58	0.63	0.59
AKOQOH	0.56	0.58	0.57	0.56	0.59	0.61	0.56	0.57	0.55	0.55
ADARUT	0.59	0.58	0.57	0.57	0.59	0.59	0.59	0.59	0.59	0.58
AFECIA	0.63	0.63	0.67	0.62	0.63	0.63	0.63	0.63	0.62	0.63
ACOVUL	0.56	0.55	0.56	0.56	0.55	0.56	0.55	0.55	0.56	0.56
AFIXEV	0.59	0.58	0.55	0.64	0.62	0.56	0.56	0.55	0.57	0.56
ABAYAF	0.60	0.62	0.62	0.62	0.63	0.61	0.62	0.62	0.62	0.62
RULJAM	0.59	0.59	0.59	0.57	0.58	0.58	0.58	0.57	0.59	0.59

S.4.1.7. ChemGE with OBabel

Task	Run									
	1	2	3	4	5	6	7	8	9	10
ABAHIW	0.67	0.70	0.67	0.68	0.72	0.68	0.69	0.67	0.67	0.67
ABAKIZ	0.71	0.72	0.70	0.73	0.76	0.71	0.70	0.71	0.71	0.71
ABADOX	0.67	0.67	0.66	0.66	0.67	0.67	0.67	0.66	0.66	0.67
ABABIP	0.68	0.67	0.67	0.66	0.68	0.68	0.70	0.67	0.68	0.67
GASQOK	0.73	0.73	0.73	0.73	0.73	0.73	0.73	0.73	0.74	0.73
ABEKIE	0.72	0.68	0.68	0.68	0.69	0.68	0.68	0.68	0.68	0.70
NIWPUE01	0.66	0.64	0.66	0.64	0.65	0.65	0.67	0.64	0.67	0.66
ABEKIF	0.57	0.57	0.57	0.57	0.57	0.57	0.57	0.57	0.57	0.57
APUFEX	0.60	0.59	0.60	0.59	0.59	0.59	0.59	0.59	0.59	0.59

ABEHAU	0.64	0.64	0.64	0.63	0.64	0.63	0.64	0.64	0.64	0.63
TITTUO	0.63	0.64	0.63	0.65	0.63	0.63	0.63	0.63	0.63	0.63
EGEYOG	0.65	0.65	0.65	0.65	0.65	0.65	0.65	0.65	0.65	0.65
ABOBUP	0.60	0.61	0.61	0.60	0.60	0.60	0.60	0.59	0.59	0.59
XIDTOW	0.73	0.69	0.72	0.72	0.73	0.69	0.70	0.67	0.73	0.68
ACNCOB10	0.66	0.69	0.65	0.65	0.67	0.67	0.66	0.66	0.66	0.66
TACXUQ	0.66	0.65	0.65	0.64	0.64	0.69	0.66	0.66	0.64	0.65
ACAZFE	0.74	0.74	0.76	0.76	0.74	0.74	0.74	0.74	0.74	0.74
NIVHEJ	0.57	0.56	0.58	0.57	0.56	0.60	0.57	0.58	0.56	0.58
ADUPAS	0.63	0.63	0.63	0.63	0.63	0.63	0.63	0.63	0.63	0.63
DAJLAC	0.68	0.66	0.67	0.67	0.67	0.67	0.67	0.68	0.67	0.66
OFOWIS	0.67	0.67	0.67	0.67	0.66	0.66	0.67	0.67	0.67	0.67
CATSUL	0.73	0.72	0.73	0.73	0.73	0.73	0.72	0.73	0.73	0.72
HESMUQ01	0.72	0.72	0.70	0.72	0.73	0.71	0.71	0.72	0.70	0.72
GUDQOL	0.62	0.64	0.63	0.63	0.64	0.65	0.63	0.62	0.66	0.62
ABEVAG	0.67	0.67	0.66	0.66	0.66	0.66	0.66	0.69	0.67	0.67
AKOQOH	0.67	0.66	0.66	0.66	0.68	0.67	0.66	0.68	0.67	0.66
ADARUT	0.62	0.60	0.61	0.62	0.60	0.62	0.61	0.61	0.60	0.61
AFECIA	0.61	0.61	0.63	0.61	0.61	0.61	0.62	0.62	0.61	0.61
ACOVUL	0.56	0.56	0.56	0.57	0.56	0.57	0.56	0.56	0.56	0.56
AFIXEV	0.67	0.67	0.67	0.66	0.67	0.67	0.67	0.67	0.67	0.67
ABAYAF	0.60	0.60	0.60	0.60	0.60	0.60	0.60	0.60	0.60	0.60
RULJAM	0.72	0.71	0.71	0.71	0.70	0.71	0.75	0.72	0.69	0.72

S.5. References

(1) Reed, A. E.; Curtiss, L. A.; Weinhold, F. Intermolecular Interactions from a Natural Bond Orbital, Donor-Acceptor Viewpoint. *Chem. Rev.* **1988**, *88*, 899–926. https://doi.org/10.1021/cr00088a005.

(2) Weinhold, F.; Landis, C. R. NATURAL BOND ORBITALS AND EXTENSIONS OF LOCALIZED BONDING CONCEPTS. *Chem. Educ. Res. Pract.* **2001**, *2*, 91–104. https://doi.org/10.1039/B1RP90011K.

(3) Green, J. C.; Green, M. L. H.; Parkin, G. The Occurrence and Representation of Three-Centre Two-Electron Bonds in Covalent Inorganic Compounds. *Chem. Commun.* **2012**, *48*, 11481–11503. https://doi.org/10.1039/C2CC35304K.

(4) King, R. B. Analogies between the Chemical Bonding Topologies in Metal–Olefin Complexes and in Metallaboranes: The Role of Three-Center Two-Electron Bonding. *J. Organomet. Chem.* **2001**, *635*, 75–83. https://doi.org/10.1016/S0022-328X(01)00789-6.

(5) Smith, H. W.; Lipscomb, W. N. Single-Crystal X-Ray Diffraction Study of β-Diborane. *J. Chem. Phys.* **1965**, *43*, 1060–1064. https://doi.org/10.1063/1.1696820.

(6) Dewar, J. A Review of the Pi-Complex Theory. *Bull. Soc. Chim. Fr.* **1951**, *18*, C71−C79.

(7) Chatt, J.; Duncanson, L. A. 586. Olefin Co-Ordination Compounds. Part III. Infra-Red Spectra and Structure: Attempted Preparation of Acetylene Complexes. *J. Chem. Soc. Resumed* **1953**, No. 0, 2939−2947. https://doi.org/10.1039/JR9530002939.

(8) Takusagawa, F.; Koetzle, T. F. A Neutron Diffraction Study of the Crystal Structure of Ferrocene. *Acta Crystallogr. B* **1979**, *35*, 1074−1081. https://doi.org/10.1107/S0567740879005604.

(9) Cole, J. C.; Korb, O.; McCabe, P.; Read, M. G.; Taylor, R. Knowledge-Based Conformer Generation Using the Cambridge Structural Database. *J. Chem. Inf. Model.* **2018**, *58*, 615−629. https://doi.org/10.1021/acs.jcim.7b00697.

(10) Yoshikawa, N.; Hutchison, G. R. Fast, Efficient Fragment-Based Coordinate Generation for Open Babel. *J. Cheminformatics* **2019**, *11*, 49. https://doi.org/10.1186/s13321-019-0372-5.

(11) Riniker, S.; Landrum, G. A. Better Informed Distance Geometry: Using What We Know To Improve Conformation Generation. *J. Chem. Inf. Model.* **2015**, *55*, 2562−2574. https://doi.org/10.1021/acs.jcim.5b00654.

(12) Allen, F. H. The Cambridge Structural Database: A Quarter of a Million Crystal Structures and Rising. *Acta Cryst B* **2002**, *58*, 380−388.

(13) Groom, C. R.; Bruno, I. J.; Lightfoot, M. P.; Ward, S. C. The Cambridge Structural Database. *Acta Crystallogr. Sect. B* **2016**, *72*, 171−179. https://doi.org/10.1107/S2052520616003954.
